# The relationships between democratic experience, adult health, and cause-specific mortality in 170 countries between 1980 and 2016: an observational analysis

**DOI:** 10.1016/S0140-6736(19)30235-1

**Published:** 2019-04-20

**Authors:** Thomas J Bollyky, Tara Templin, Matthew Cohen, Diana Schoder, Joseph L Dieleman, Simon Wigley

**Affiliations:** a Council on Foreign Relations, Washington, DC, USA; b Department of Health Research and Policy, Stanford University School of Medicine, Stanford, CA, USA; c Institute for Health Metrics and Evaluation, University of Washington, Seattle, WA, USA; d Department of Philosophy, Bilkent University, Ankara, Turkey

## Abstract

**Background:**

Previous analyses of democracy and population health have focused on broad measures, such as life expectancy at birth and child and infant mortality, and have shown some contradictory results. We used a panel of data spanning 170 countries to assess the association between democracy and cause-specific mortality and explore the pathways connecting democratic rule to health gains.

**Methods:**

We extracted cause-specific mortality and HIV-free life expectancy estimates from the Global Burden of Diseases, Injuries, and Risk Factors Study 2016 and information on regime type from the Varieties of Democracy project. These data cover 170 countries and 46 years. From the Financing Global Health database, we extracted gross domestic product (GDP) per capita, also covering 46 years, and Development Assistance for Health estimates starting from 1990 and domestic health spending estimates starting from 1995. We used a diverse set of empirical methods—synthetic control, within-country variance decomposition, structural equation models, and fixed-effects regression—which together provide a robust analysis of the association between democratisation and population health.

**Findings:**

HIV-free life expectancy at age 15 years improved significantly during the study period (1970–2015) in countries after they transitioned to democracy, on average by 3% after 10 years. Democratic experience explains 22·27% of the variance in mortality within a country from cardiovascular diseases, 16·53% for tuberculosis, and 17·78% for transport injuries, and a smaller percentage for other diseases included in the study. For cardiovascular diseases, transport injuries, cancers, cirrhosis, and other non-communicable diseases, democratic experience explains more of the variation in mortality than GDP. Over the past 20 years, the average country's increase in democratic experience had direct and indirect effects on reducing mortality from cardiovascular disease (−9·64%, 95% CI −6·38 to −12·90), other non-communicable diseases (−9·14%, −4·26 to −14·02), and tuberculosis (−8·93%, −2·08 to −15·77). Increases in a country's democratic experience were not correlated with GDP per capita between 1995 and 2015 (ρ=–0·1036; p=0·1826), but were correlated with declines in mortality from cardiovascular disease (ρ=–0·3873; p<0·0001) and increases in government health spending (ρ=0·4002; p<0·0001). Removal of free and fair elections from the democratic experience variable resulted in loss of association with age-standardised mortality from non-communicable diseases and injuries.

**Interpretation:**

When enforced by free and fair elections, democracies are more likely than autocracies to lead to health gains for causes of mortality (eg, cardiovascular diseases and transport injuries) that have not been heavily targeted by foreign aid and require health-care delivery infrastructure. International health agencies and donors might increasingly need to consider the implications of regime type in their efforts to maximise health gains, particularly in the context of ageing populations and the growing burden of non-communicable diseases.

**Funding:**

Bloomberg Philanthropies and the Bill & Melinda Gates Foundation.

## Introduction

Democratic governance has not been a driving force in global health. Many of the countries that have had the greatest improvements in life expectancy and child mortality over the past 15 years are electoral autocracies that achieved their health successes with the heavy contribution of foreign aid. Ethiopia, Myanmar, Rwanda, and Uganda all extended their life expectancy by 10 years or more between 1996 and 2016.^1^ The governments of these countries were elected in multiparty elections designed so only the opposition could lose. Consequently, these countries rank in the bottom third of countries on the University of Gothenburg's Varieties of Democracy (V-Dem) Index,^2^ making them among the least democratic nations in the world. In 2016, these countries were among the top two dozen recipients of development assistance for health.^3^ Although many bilateral aid agencies emphasise the importance of democratic governance in their policy statements, most studies of development assistance have found no correlation between foreign aid and democratic governance and, in some instances, a negative correlation.^4^, ^5^, ^6^, ^7^ In response to criticism of President Paul Kigame's 2017 re-election with 98·7% of the vote, the former Rwandan health minister argued that “the true measure of democracy is not elections, but education, health, and security for the people”.^8^

**Research in context**

**Evidence before this study**

Over the past 25 years, various cross-country studies have assessed whether democracy is related to improved population health. Almost all of these studies have limited their assessments to democracy's effect on mortality of infants and children younger than 5 years, or life expectancy at birth, primarily reflecting gains in child and infant health. The results of these studies largely support the notion that democracy is associated with better population health, but at least four studies have found no link between democracy and infant or child mortality. Critics of the methodological shortcomings of these democracy and health studies attribute the improved health outcomes to factors such as institutional capacity and country income, rather than regime type. Most studies of the allocation of development assistance to national governments have found that it remains uncorrelated with democratic governance and, in some instances, negatively correlated.

To identify studies that have investigated the relationship between democracy and population health, we searched, without date restrictions, Scopus, Google Scholar, and PubMed for all articles with “democracy” and “health” or “mortality” in the abstract or title. To the best of our knowledge, no other studies comprehensively assess the global effect of democratic governance on non-communicable diseases and injuries—health concerns that represent an increasing share of the disease burden in low-income and middle-income countries.

**Added value of this study**

This study brings updated, expanded, and improved data sources, a diverse set of methodologies, and a focus on non-communicable diseases, injuries, and adult health to the exploration of the relationship between democracy and health. To characterise the effect of democratic experience on country health burden, we leveraged estimates from the Global Burden of Diseases, Injuries, and Risk Factors Study (GBD) 2016, the University of Gothenburg's Varieties of Democracy project, and the Financing Global Health database. These data capture detailed political, economic, and population health information and enable us to construct a panel of data spanning 170 countries to assess the role of democracy, and its core components, on HIV-free life expectancy at age 15 years, cause-specific mortality, and health spending, and on the pathways connecting democratic rule to cause-specific health gains. In addition to broad population health measures, we used disaggregated cause-specific mortality estimates from GBD 2016.

**Implications of all the available evidence**

Democratic governments have a greater incentive to improve adult health and reduce mortality from non-communicable diseases and injuries than their autocratic counterparts. These results are consistent with the perception that democracies are more apt than autocracies to implement proven treatment and prevention interventions for causes not heavily targeted by foreign aid and requiring health-care delivery infrastructure. Regular free and fair elections appear important for improving adult health and non-communicable disease outcomes, most likely by increasing government accountability. This study suggests that democratic institutions and processes might help to enhance and expand the effectiveness of global health programmes and initiatives, especially with regard to non-communicable diseases, injuries, and adult health.

The theoretical reasoning that democracy should improve population health is straightforward. First, when enforced through regular, free, and fair elections, democracies should have a greater incentive than autocracies to provide health-promoting resources and services to a larger proportion of the population.^9^ Second, democracies are more open to feedback from a broader range of interest groups, more protective of media freedom, and might be more willing to use that feedback to improve their public health programmes. Autocracies reduce political competition and access to information, which might deter constituent feedback and responsive governance.^10^

Various studies^11^, ^12^, ^13^, ^14^, ^15^ have concluded democratic rule is better for population health; almost all focus on infant and child mortality or life expectancy at birth. Some academics^16^ have questioned those studies' results, arguing that democratic leaders do not need the electoral support of low-income voters to stay in office. Others^17^ have claimed that the underlying determining factor is wealth or the quality of government institutions, rather than the democratic process. At least four studies^16^, ^18^, ^19^, ^20^ have found that democracy has no clear relationship with child and infant mortality.

Assessments of the role of democratic governance in child and infant health might not be generalisable to non-communicable diseases, which are largely chronic, require more health delivery infrastructure and skilled workforces, and are costlier to treat than many communicable diseases.^21^ Global health donors and intergovernmental institutions have also historically prioritised communicable diseases, and infant, child, and maternal mortality over non-communicable diseases and injuries in their programmes. Without the same external pressure or validation from donors to do more to address non-communicable diseases and injuries, autocratic leaders have less incentive than their democratic counterparts to finance their prevention and treatment. In democracies, the political survival of leaders is more likely to hinge on maintaining capable health systems and reducing premature death and disability across all age groups.

Autocracies, such as in Cuba and China, known for providing good health at low cost, have not always been as successful when their populations' health needs shift to non-communicable diseases. A 2017 assessment^22^ found that observed life expectancy in China was lower than its expected life expectancy at birth, on the basis of its Socio-demographic Index, from 1980 to 2000, and it has improved only over the past decade with increased government health spending. The degree to which Cuba's observed life expectancy has exceeded expectations has decreased, from 4·7 years higher than expected in 1970, to 3·5 years higher than expected in 2016.

To the best of our knowledge, there is no robust research on the role of democracy in adult health and the burden of non-communicable diseases and injuries. Safaei's^23^ and Mackenbach and colleagues'^24^ studies are the closest examples; however, Safaei considers a single year of data (2002–03) for adult mortality and Mackenbach and colleagues only assess mortality from some non-communicable causes in European nations before 2008, and the methods of both studies have limitations, making them unable to draw global health policy implications.

A comprehensive reassessment of the role of democracy in global health is past due. In this study, we used a panel of data spanning 170 countries to examine the role of democracy in population health and the pathways connecting democratic rule to health gains. We also explore the relationships between democracy and cause-specific mortality and with life expectancy at age 15 years. These results are important for determining whether promotion of accountable and open democratic institutions should play a greater role among the portfolio of international accountability mechanisms being pursued to improve population health outcomes and to increase investment in high-quality, accessible health care. This evidence is timely as UN member states and civil society groups consider how to build on the momentum and commitments made in the 2018 high-level meetings on non-communicable diseases^25^ and tuberculosis.^26^

## Methods

### Approach

Our data sources included the Global Burden of Diseases, Injuries, and Risk Factors Study (GBD) 2016,^1^ V-Dem,^2^ and Financing Global Health databases.^3^ These data capture detailed information about country political and economic context, and population health. Our empirical strategy was to leverage multiple statistical techniques, each testing a different element of the relationship, and use the strengths of each to assess the overall effect of democratic experience on health. We used the synthetic control method, which compares observed data for countries undergoing democratic transition to the constructed counterfactual scenarios; a fixed-effects regression to measure variation in annual cause-specific death rates; a structural equation model to assess potential pathways by which democratisation might improve health; and a fixed-effects regression to do a sensitivity analysis, systematically removing components of the democracy measure to understand which component was most associated with population health improvements. Together, we believe this diverse set of methods form a robust analysis of the governance–population health relationship.

### Data

We extracted cause-specific mortality and HIV-free life expectancy from the GBD 2016 database.^1^ The GBD project estimates age-specific and sex-specific mortality for 264 causes of death from 1980 to 2016 (death) and 1970 to 2016 (life expectancy) in 195 countries. We used age-standardised and sex-standardised death rates, aggregated to the GBD Level 2 cause, which included 21 causes of illness groups, such as cancer and cardiovascular diseases. HIV-free life expectancy estimates life expectancy in each country had the HIV pandemic not occurred, excluding war and natural disasters. These methods have been previously described.^27^

The V-Dem database provides numerous indicators of regime characteristics, such as electoral fraud, multiparty elections, freedom of civil association, and media freedom, for 201 countries from 1789 to 2017.^2^ Each indicator was constructed on the basis of coding by multiple country experts. Patterns of agreement or disagreement between experts were used to estimate, and thereby correct for, measurement error. We used V-Dem's Multiplicative Polyarchy Index, created by multiplying the five core components of electoral democracy (ie, suffrage, free and fair elections, elected officials, freedom of civil and political organisation, and freedom of expression) to construct a democratic experience variable. We calculated democratic experience by taking the sum of each country's index score from 1900 to the observation year. We used accumulated stock of democracy, rather than level of democracy at each point in time, to capture the proximal and distal effects of policies resulting from democratic reform, as described in detail in the appendix.^12^ The interventions required to reduce premature death due to non-communicable diseases will often take an extended period of time to have an effect. To account for the diminishing effect of policies implemented even further back in time, a modest annual depreciation of 1% was applied to the stock variable, as has been done in previous studies.^12^ In this study, we refer to our variable as democratic experience.

We extracted government health expenditure as source and development assistance for health from the Institute for Health Metrics and Evaluation Financing Global Health 2017 database.^3^, ^28^, ^29^ This database includes health spending estimates for 188 countries from 1990 to 2015. We define government health spending as country government spending on health, exclusive of development assistance.

The GBD Covariate Database^30^ assembles numerous covariates that are relevant to health, most of which are available for 195 countries from 1980 to 2016. We used mean years of education, mortality shocks, and gross domestic product (GDP) per capita. Mean years of education captured the educational attainment by age and sex from multiple surveys, such as the Demographic and Health Surveys. Mortality shocks captured the increase in death rates due to famine and war.^31^ We complement these covariates with the percentage of the population living in urban areas from the World Development indicators.^32^ GDP per capita was based on four commonly used GDP per capita series: World Bank World Development Indicators,^32^ International Monetary Fund World Economic Outlook report,^33^ Angus Maddison's research homepage at the University of Groningen,^34^ and the University of Pennsylvania Center for International Comparisons of Production, Income, and Prices.^35^ Each of the four series is imputed separately with growth regressions and then averaged. These methods have been previously described.^36^ GDP per capita is reported in 2017 purchasing power parity-adjusted dollars.^32^, ^33^, ^34^, ^35^

### Statistical analysis

Our first method examined whether or not countries that underwent a democratic transition subsequently had increased HIV-free life expectancy at age 15 years. The synthetic control method estimates the effect of democratisation on life expectancy by comparing trends in the life expectancy of countries that transitioned from autocracy to democracy and the weighted average of 55 countries that remained entirely autocratic. The method has two main advantages: it relies on a robust and replicable process to select comparison countries; and it reduces concerns about time-varying confounders because of how the synthetic control is constructed.^37^

To complete this synthetic control method analysis, we identified 15 countries that had an unambiguous democratic transition from 1980 to 2000, and stayed democratic through 2015, at least. These 15 countries received this classification if they increased to a score of one on the ordinal version of V-Dem's Multiplicative Polyarchy Index. Next, synthetic controls, which are the weighted average of the 55 countries that remained entirely autocratic from 1970 to 2015, were created for each of the transitioning countries. Weights were set such that the weighted average of the continuously autocratic countries matched each transitioning country in terms of characteristics such as urbanicity (the effect of living in an urban area), educational attainment, GDP per capita, and child mortality. The 15 transitioning and 55 continuously autocratic countries and country characteristics are listed in the appendix. We estimated the effect of democratisation by comparing the average HIV-free life expectancy at age 15 years for the transitioning countries with the average HIV-free life expectancy at age 15 years for the synthetic controls. Significance of effect was determined by a permutation test, which empirically tests that no treatment effect exists in autocratic states, rather than using asymptotics to determine CIs.^38^

HIV-free life expectancy was used for this analysis to isolate and assess the effect of regime type on adult health. Unlike other causes that disproportionately affect adult health, democratic and autocratic countries alike received substantial amounts of external development assistance for HIV/AIDS. At least ten of the 15 democratic transition countries, and 47 of 55 continuously autocratic countries, assessed received substantial annual amounts of international development assistance for HIV/AIDS for some or all of our study period.^39^

Our second analysis examined how democratic experience, national income, development assistance for health, urbanicity, and mortality shocks explain changes in mortality. We used a Shapley Variance Decomposition to estimate the fraction of the within-country variance of age-standardised mortality explained by each determinant. To assess within-country variation, we used a country fixed-effect regression, which controls for the fact that countries have unique unobserved characteristics. We regressed mortality on our selected determinants of health, and country and year fixed-effects. Year fixed-effects captured the effect of non-linear changes across time that affect all countries in a given year.

Our third analysis investigated the pathways by which democratic experience might improve health and the magnitude of that improvement. We used a structural equation model to represent these pathways and estimate the direct and indirect effect of democracy on health. Structural equation modelling is a method that simultaneously estimates multiple linear regressions to assess the direct association that a variable might have on an outcome, and the indirect pathways by which that key covariate might affect other covariates, which ultimately affect the outcome. We modelled changes in mortality as a function of changes in democratic experience, GDP per capita, mean years of education, urbanicity, skilled birth attendance, and government health expenditure. In addition to having their own direct effects, GDP per capita, government health expenditure, and urbanicity were treated as potential mediating (indirect) pathways by which democratic experience might improve health. The structural equation model and a pathway diagram illustrating our equation structure are included in the appendix.

To make our results more actionable to policy makers, we did an analysis to identify which components of democratic experience are most associated with population health improvements, using the linear regression specification from analysis 2. We systematically removed each of the components of democracy from the democracy stock index and re-estimated the regression. The components sequentially removed were free and fair elections, suffrage, freedom of association, freedom of expression, and elected executive. This leave-one-out strategy tests the hypothesis that, although the components of democratic experience are intertwined, one might be particularly strongly associated with mortality. Further sensitivity analyses are presented in the appendix.

All analyses were done with Stata, version 15.1, and R, version 3.4.3.

### Role of the funding source

We received funding from Bloomberg Philanthropies and the Bill & Melinda Gates Foundation, who had no role in data collection, analysis, or interpretation, or any aspect pertinent to the study. All authors had full access to all the data in the study and had final responsibility for the decision to submit for publication.

## Results

**Figure 1:**
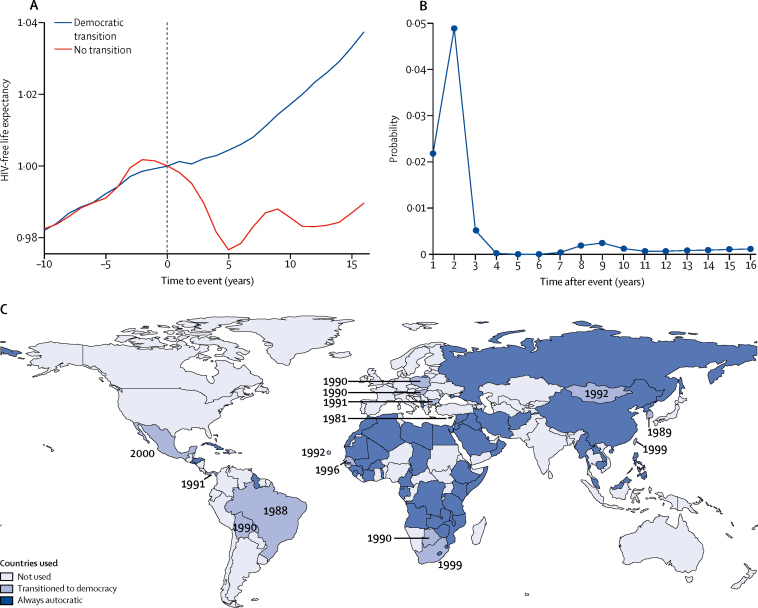
The average effect of democratic transition on HIV-free life expectancy at age 15 years (A) The average effect on HIV-free life expectancy due to a democratic transition over 15 years. The blue line represents the normalised life expectancy in the 15 countries that underwent a democratic transition. The red line represents the normalised life expectancy in the corresponding synthetic countries that did not undergo a democratic transition. Year 0 is when the transition took place. Divergence after year 0 reflects the estimated average effect of democratic transitions. Life expectancy normalised to 1 in year 0. (B) The results of an empirical permutation test to determine if countries randomly might experience this improvement in HIV-free life expectancy. A low probability indicates that this improvement in life expectancy is unlikely to have happened by chance. (C) Democratising countries used in the synthetic control analysis are marked in blue, with year of transition noted. Continuously autocratic countries are in navy and countries that previously democratised or reverted back to autocracy are in light blue.

Our first analysis showed that, controlling for HIV/AIDS, the average life expectancy at age 15 years increased after 10 years in the countries that underwent a democratic transition by 3% (p=0·001), relative to the synthetic counterfactual of no transition (figure 1). The improvement in adult health after the transition to democracy is immediate (an average of 0·3% in the first year) and continues to build over time. The improvements are statistically different from the counterfactual of no democratisation from the first year after the transition (p=0·02; figure 1).

**Figure 2:**
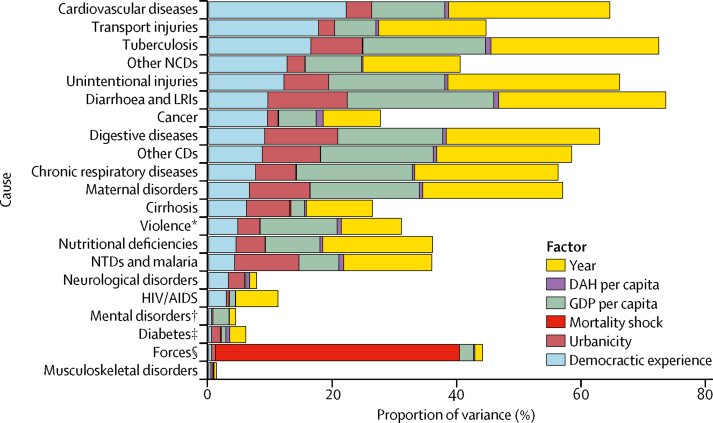
Changes in mortality due to democracy and other determinants of health The proportion of variance in Global Burden of Disease Level 2 cause-specific, age-standardised mortality explained by democracy and other determinants of health such as gross domestic product (GDP) per capita, urbanicity, development assistance for health (DAH), and mortality shocks such as war. The sum of the variance explained by each variable is the *r*^2^, which is the share of the variance of mortality explained by the model. NCDs=non-communicable diseases. LRIs=lower respiratory and other common infectious diseases. CDs=communicable diseases. NTDs=neglected tropical diseases. *Includes self-harm and interpersonal violence. †Includes substance use disorders. ‡Includes diabetes, urogenital, blood, and endocrine diseases. §Includes forces of nature, conflict, terrorism, and state violence.

**Figure 3:**
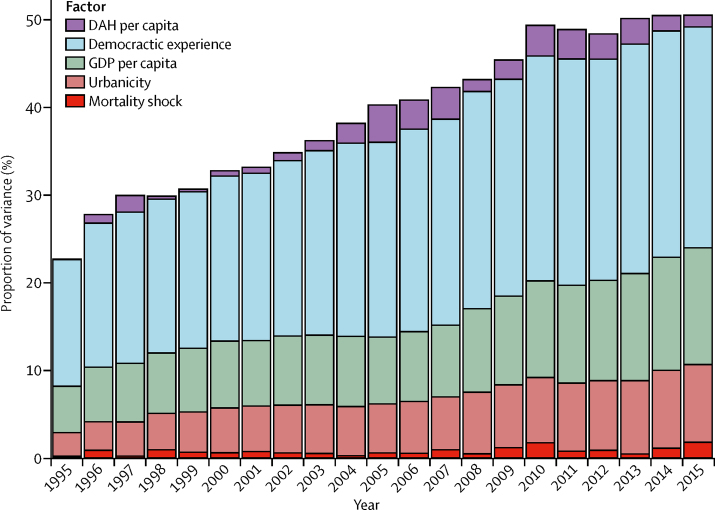
Changes in cardiovascular disease across time The proportion of explained variance in age-standardised cardiovascular disease death rates across 188 countries from 1995 to 2015. DAH=development assistance for health. GDP=gross domestic product.

Our second analysis found that democratic experience explained the largest portion of the variation in mortality for cardiovascular diseases (22·27%) and transport injuries (17·78%): nearly a quarter of country-specific variation (figure 2). Democratic experience explains more of the variation in mortality within country than GDP for cardiovascular diseases (22·27% *vs* 11·83%), transport injuries (17·78% *vs* 6·65%), cancers (9·50% *vs* 6·07%), cirrhosis (6·14% *vs* 2·18%), and other non-communicable diseases (12·68% *vs* 9·14%), such as congenital heart disease and congenital birth defects. The importance of democratic experience in explaining the variation in mortality from cardiovascular diseases and transport injuries within a country has increased over time, from 14·40% in 1995 to 25·23% in 2015 for cardiovascular diseases (figure 3) and from 22·12% to 28·12% for transport injuries (appendix) over the same period. However, these factors combined still explain only 50·48% (39·29% on average across all years, ranging from 22·65% in 1995 to 50·48% in 2015) of the total observed variance for cardiovascular disease in 2015. Further, democratic experience explained little of the variation in the mortality within a country from some leading communicable causes of death such as HIV (2·82%) and malaria and neglected tropical diseases (4·18%), but also did not explain much of the variation in mortality from diabetes (0·44%), mental health (0·45%), or musculoskeletal disorders (0·33%; figure 2).

**Figure 4:**
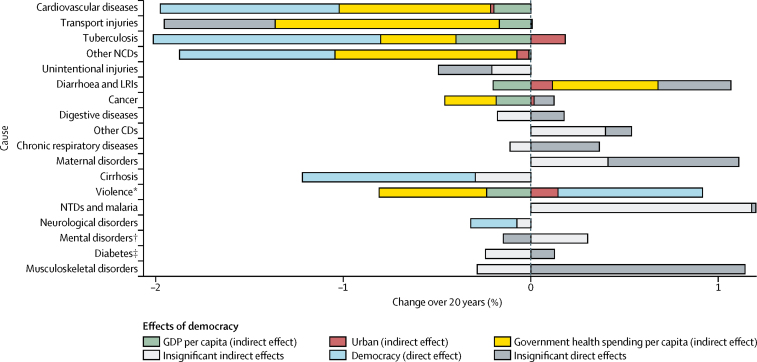
Long-term effect of democracy on country disease burden The estimated direct and indirect long-term effects of democracy on health from a structural equation model. The blue bars show the direct effect of democratic change on changes in health. The green, red, and yellow bars show the indirect effects of democratic change on changes in health due to resulting changes in gross domestic product (GDP) per capita, urbanicity, and government health expenditure as source. All changes are from 1995 to 2015. Results that were not statistically significant are shown in grey. HIV/AIDS and forces were omitted because of distortion of the x-axis of the graph, such that the other causes were not visible; all results are available in the appendix. NCDs=non-communicable diseases. LRIs=lower respiratory and other common infectious diseases. CDs=communicable diseases. NTDs=neglected tropical diseases. *Includes self-harm and interpersonal violence. †Includes substance use disorders. ‡Includes diabetes, urogenital, blood, and endocrine diseases.

Our third analysis identified the pathways by which democratic experience is associated with changes in cause-specific mortality and estimates the magnitude of those associated effects. Those effects can be direct, via the effect of democratic experience itself, and indirect, through the effect of democratic experience on other measurable factors, such as increased government health spending and economic growth, which in turn might affect mortality. Our results show that a one-point increase in democratic experience had significant direct and indirect effects on reducing mortality over 20 years from cardiovascular disease (−1·97%, 95% CI −1·31 to −2·64), other non-communicable diseases (−1·87%, −0·87 to −2·87), including congenital heart diseases and birth defects, and tuberculosis (−1·83%, −0·42 to −3·23; figure 4). Democracy also had significant indirect effects on mortality over 20 years from transport injuries (−1·94%, −0·90 to −2·99). Government health expenditure and GDP per capita were the primary indirect pathways by which that 20-year mortality reduction occurred for cardiovascular diseases (−0·81% [–1·22 to −0·40] and −0·20% [–0·40 to 0·00], respectively), transport injuries (−1·19% [–1·82 to −0·57] and −0·18% [–0·46 to 0·12], respectively), and tuberculosis (−0·40% [–1·6 to 0·26] and −0·40% [–0·85 to 0·05], respectively); the indirect effects of democracy for other non-communicable diseases were mostly limited to government health expenditure (−0·97%, −1·54 to −0·40). The median country observed a 4·88-point increase in democratic experience from 1995 to 2015 (appendix).

Government health expenditure is also the pathway by which democratic experience had a modest, but significant, indirect effect on reducing mortality from cancers (−0·44%, 95% CI −0·69 to −0·19) and violence (−0·66%, −1·13 to −0·19). Democratic experience had a modest direct effect on reducing mortality from cirrhosis (−0·92%, −1·83 to −0·01), and neurological disorders (−0·25%, −0·52 to 0·03), and on increasing deaths from violence (0·77%, −0·12 to 1·66). Democratic experience had an indirect effect on increasing mortality from diarrhoeal diseases (0·47%, 0·00 to 0·95), but that result might be misleading. Mortality from diarrhoeal diseases has declined dramatically in poor non-democracies relative to the already very low mortality from that cause in wealthy democratic nations. Democratic experience did not have a statistically significant total effect on mortality from HIV (−1·77%, −61·80 to 58·26), other communicable diseases (0·54%, −0·51 to 1·59), digestive disease (−0·00%, −0·74 to 0·73), unintentional injuries (−0·49%, −1·14 to 0·16), respiratory diseases (0·54%, −0·51 to 1·59), maternal diseases (0·70%, −0·67 to 2·07), mental disorders (0·16%, −1·50 to 1·81), musculoskeletal disorders (0·86%, −0·60 to 2·31), or diabetes (−0·12%, −1·24 to 1·00).

**Figure 5:**
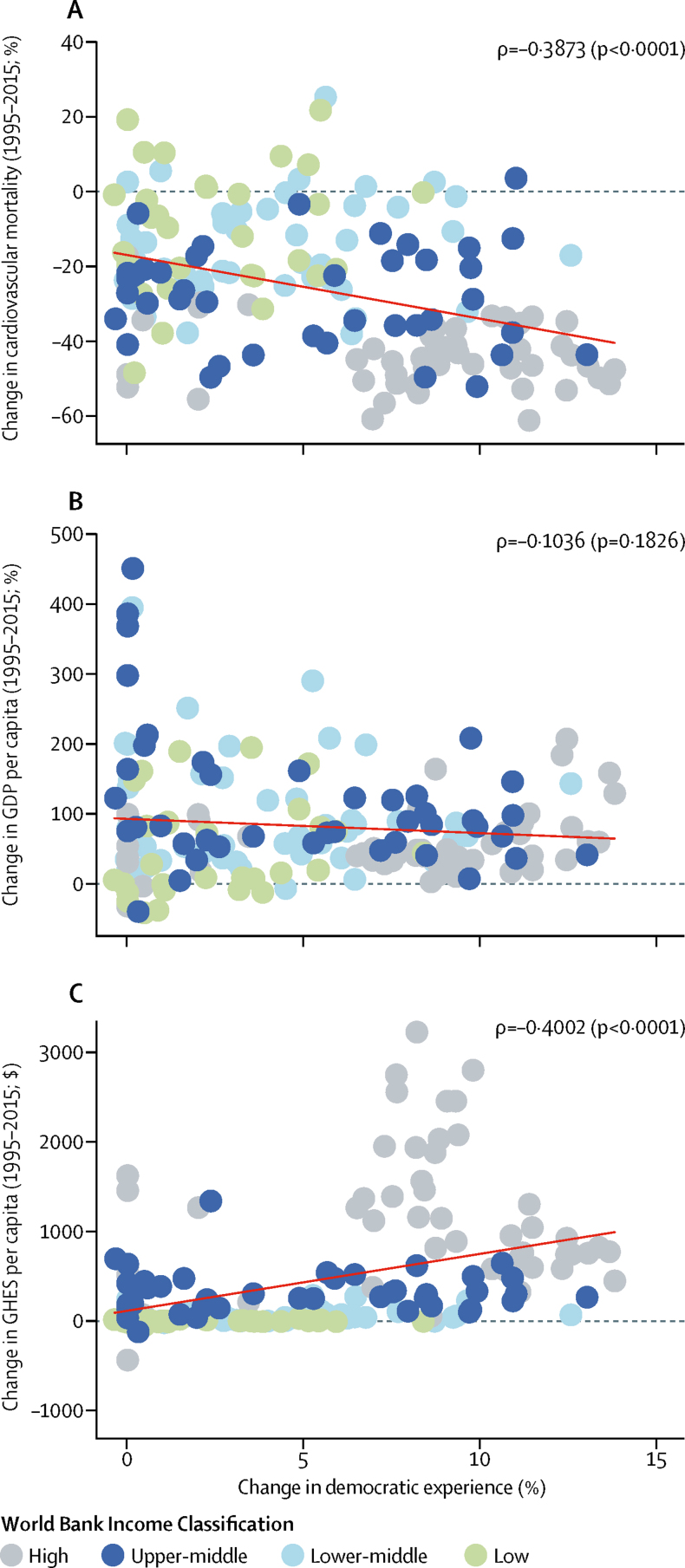
Cross-country changes in democracy, cardiovascular disease, and health spending The relationships between changes in democracy and cardiovascular age-standardised death rates (A); changes in democracy and growth in gross domestic product (GDP) per person (B); changes in democracy and government health expenditure (GHES) as source (C). All panels show changes from 1995 to 2015. The red line is a linear fit through the bivariate relationship.

The results of our fixed-effect and structural equation models were reinforced by the correlations that we found with changes in democratic experience and GDP per capita, government health expenditure, and cardiovascular mortality. Our analysis showed that changes in democratic experience from 1995 to 2015 have an ambiguous bivariate relationship with changes in GDP per capita (ρ=–0·1036; p=0·1826), but democratic experience is positively correlated with government health expenditure (ρ=0·4002; p<0·0001) and inversely related to cardiovascular mortality (ρ=–0·3873; p<0·0001; figure 5). For high-income nations, the relationship between government health spending and increases in democratic experience was more ambiguous (ρ=0·0855; p=0·5593). Correlations by income group are presented in the appendix.

**Figure 6:**
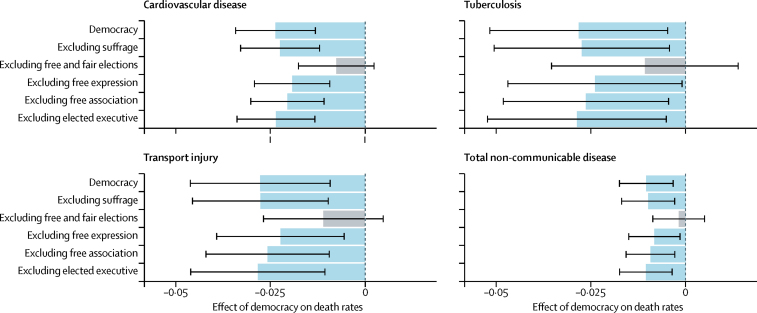
Critical component of democracy for four causes The average effect of democracy on non-communicable disease, cardiovascular disease, tuberculosis, and transport injury age-standardised death rates using the leave-one-out strategy. The effect is measured as the percent change in death rates associated with a one-unit increase in democratic experience. The black lines indicate the 99% CI on the effect size. Blue bars indicate significant effects on health, whereas grey bars are insignificant (significance determined at p=0·01).

Our fourth analysis shows that removing free and fair elections from the democratic experience variable resulted in the negative association between that variable and age-standardised mortality from cardiovascular diseases, transport injuries, tuberculosis, and total non-communicable diseases no longer being statistically significant (p=0·052, p=0·075, p=0·263, and p=0·497, respectively; figure 6). By contrast, removal of each of the other elements of the democratic experience index—suffrage, free expression, freedom of association, and elected executives—did not significantly alter the association between democracy and age-standardised mortality from these causes. The results for all causes are given in the appendix.

## Discussion

Our research indicates that democratic experience matters for global health. It is likely to matter more as the dominant concerns of countries continue to shift from communicable diseases and child and infant health to non-communicable diseases, injuries, and the health needs of adults. We draw four conclusions from our analysis.

First, policy makers, donors, and international institutions concerned with premature death and disability in low-income and middle-income nations should also be concerned with democratic experience. In 2016, the four causes for which democratic experience matters most—cardiovascular diseases, tuberculosis, transport injuries, and other non-communicable diseases—caused 25·3% of the total death and disability in individuals younger than age 70 years in low-income and middle-income countries. That same year, cardiovascular diseases—the cause for which democratic experience explained the most variation in mortality within country—accounted for 14 million deaths in low-income and middle-income countries, 41·6% of which occurred in individuals younger than age 70 years. Our results show that a one-point increase in democratic experience reduced deaths by roughly 2% from cardiovascular diseases, tuberculosis, transport injuries, and other non-communicable diseases.

Second, our results are consistent with the conclusion that countries with more democratic experience were more apt than autocracies to make health gains for those causes that require quality health care and government policy-based prevention, and are not heavily targeted by development assistance for health. The hypothesis underlying this conclusion is that the direct effects of democratic experience are that governments are more open to feedback from interest groups and to constituents sharing health-care information, more protective of media freedom, and more willing to use that feedback to improve the quality of health-care services. The indirect effects of democracy are expected to be measurable in terms of factors such as increased government health spending. Our results are consistent with this hypothesis.

The causes most affected by democratic experience are more dependent on chronic care and government policy-based prevention than other infectious, maternal, and neonatal diseases. Low-cost medicines and evidence-backed management and control strategies exist for hypertension, which causes most of the death and disability from coronary heart disease and stroke worldwide.^40^, ^41^ Hypertension management does require, however, diagnosis, financing, a health workforce, procurement and supply chain management of quality medicines, and information systems. Chemotherapy for tuberculosis is one of the most cost-effective of all health interventions, but it also necessitates use of multiple drugs over an extended time.^42^ Like hypertension, effective tuberculosis control requires enforcement of standard diagnostic and treatment protocols; a consistent supply of essential quality-assured medicines; identification of at-risk individuals participating in other health-care services; and systematic monitoring and evaluation of outcomes and patients. Tobacco control reduces the risks of cardiovascular disease and tuberculosis infection alike, but requires governments to implement and enforce excise taxes, smoke-free laws, and advertising restrictions. Transport injuries can be cut with enforcement of laws against driving without motorcycle helmets or seatbelts; while intoxicated; or at excessive speeds.^43^ Investment in trauma care and surgery can improve the outcome of transport injuries, congenital heart disease, and congenital birth defects.

The causes most affected by democratic experience are generally targeted less by international donors, relative to other infectious, maternal, and neonatal diseases. Non-communicable diseases (US$825 million in development assistance for health in 2017) and injuries are not targeted with development assistance for health commensurate with their burden. Tuberculosis is a leading cause of communicable disease deaths globally, but fewer donor resources ($1·76 billion in 2017) are devoted to its prevention and treatment than to HIV/AIDS ($9·21 billion), malaria ($2·58 billion), and for childhood vaccinations that tend to focus on diarrhoeal and lower-respiratory infections ($2·63 billion).^3^ Without the same resources, external pressure, or validation from donors, autocratic leaders might have less incentive to provide the chronic care and policy-based prevention that tuberculosis, injuries, and many non-communicable diseases require. Our results show that governments, irrespective of GDP, spent more on health as democratic experience increased, which is consistent with the findings of previous studies.^44^, ^45^

Our results are consistent with the findings of the recent *Lancet Global Health* Commission on High Quality Health Systems,^46^ which found the effect of health-care quality on mortality in 137 low-income and middle-income countries was highest for the same set of causes most affected by democratic experience: cardiovascular diseases, road injuries, tuberculosis, and neonatal deaths, which include congenital diseases.^47^ In the Commission's analysis,^46^ the consequences of paucity of quality health care on these causes were also much larger than for communicable and maternal diseases. Cardiovascular diseases alone were responsible for 2·36 million of the 5·04 million deaths from the absence of quality health care in low-income and middle-income countries in 2016.^47^

Similar factors might explain the modest, but significant association between democratic experience and mortality for other non-communicable causes not targeted by development assistance for health and requiring quality health-care delivery systems. Earlier detection, more accurate diagnosis, and more widely available basic treatment can significantly reduce mortality from many cancers, but it is the primary responsibility of governments to ensure universal, affordable access to cancer care. Most countries have much more to do. The modest effects of democratic experience might reflect the fact that deaths from many cancers are not as amenable to treatment or as preventable as are mortality from cardiovascular diseases, tuberculosis, and transport injuries. For example, cardiovascular age-standardised death rates differ vastly by country, from 95 per 100 000 in Japan to 830 per 100 000 in Afghanistan. Similarly, age-standardised deaths from tuberculosis range from 483 deaths per 100 000 in the Central African Republic to 0·21 per 100 000 in the USA. Age-standardised death rates for most cancers do not differ internationally this much, although there are important exceptions. Effective cancer prevention measures exist, such as tobacco control and vaccines for infection-caused cancers, but prevention is less effective for many common cancers like breast and prostate, leukaemia, non-Hodgkin lymphoma, and paediatric cancers.^48^ The direct effect of democratic experience on reducing cirrhosis deaths might reflect the decline in excessive alcohol consumption in some eastern and central European states after the fall of the Soviet Union.

Democracy does not have significant effects on mortality from all non-communicable diseases, and the reasons might be cause specific. There are effective diabetes treatments and blood glucose monitoring tools, but diabetes cases are increasing internationally in poor and wealthy countries alike, because of increases in obesity, physical inactivity, and other risk factors. Democratic experience might improve quality care, but that would do little to address deaths from chronic respiratory illnesses and diabetes that are largely driven by non-utilisation of health-care services.^46^, ^47^ Low death rates for musculoskeletal, mental, and neurological disorders (on which democracy does have a small direct effect) make our results on those causes harder to parse.^49^, ^50^

Third, free and fair elections appear important for improving adult health and non-communicable disease outcomes, most likely by increasing government accountability and responsiveness. The elements of democracy—which also include suffrage, freedom of association, freedom of expression, and an elected executive—interact and work synergistically, but our results suggest free and fair elections might be essential for the health-promoting effects of democracy. Free and fair elections force governments to answer to a broad set of citizens, at regular intervals, for their adoption of proven treatment and prevention interventions.^14^ This might be the reason that democratic experience is increasingly important for cardiovascular mortality. Democracies have done a better job of adopting strategies that previously cut US cardiovascular disease death rates by more than 40% between 1980 and 2000, and reduced stroke and coronary heart disease by two-thirds in some high-income countries.^51^, ^52^ By contrast, autocracies are more likely to be answerable only to smaller groups such as the military and business interests. Autocracies might also be more likely to withhold health and welfare services from the supporters of opposition groups.^53^ In poorer districts, the government might find it more cost-effective to buy votes with small gifts and payments, rather than to win support by providing public services that will have a more lasting effect on population health.^54^ We estimate that in 2016, vote buying was common practice in 55% of the low-income and middle-income countries that have elections.^2^ As such, vote buying and electoral fraud are two areas where democratic reform could potentially have a positive effect on population health.

Fourth, these results have important implications for the allocation of development assistance for health, especially at a time when the disease burden is shifting rapidly from communicable to non-communicable diseases in many low-income and middle-income nations.^55^ The growth in development assistance for health has largely flatlined since 2011. Only 2% of the total development assistance for health in 2016 was devoted to non-communicable diseases, which represented 58% of the death and disability in low-income and middle-income countries that same year. There are no obvious indications that development assistance for health is likely to increase in general or for non-communicable diseases specifically. In this context, it is more important than ever to consider where and how to spend development assistance for health more effectively.

Multiple initiatives during 2018 have advocated measures such as human capital indices to increase country accountability as a means of improving government health spending, strengthening the global quality of health-care services, and implementation and investment of proven prevention and treatment measures for tuberculosis and non-communicable diseases.^46^, ^56^, ^57^, ^58^, ^59^, ^60^, ^61^, ^62^ There is widespread agreement that progress on the global health challenges that loom largest will need to be country-led and that success will depend on political will, accountability, and transparency. The results of this study suggest that democratic experience is one way in which that political will, accountability, and transparency might be improved and country-led progress on non-communicable diseases, tuberculosis, and adult health might ensue.

In light of these findings, one option for proceeding is increasing the funding for the development agency-led programmes for democracy promotion and governance that already exist in many European nations, the USA, and Canada, and their variants at the World Bank and other intergovernmental institutions.^63^, ^64^ These programmes seek to help countries to strengthen their democratic processes and build more accountable institutions, and many have existed for more than two decades and have operated in dozens of countries. These programmes have been lightly funded in recent years outside of active war zones.^64^, ^65^ Another option would be directing more of the scarce development assistance for health for causes where democratic experience matters, such as cardiovascular diseases, tuberculosis, or transport injuries, to the nations that have shown a commitment to building accountable institutions and open and transparent democratic processes. The US Millennium Challenge Corporation and Swedish International Development Cooperation Agency have used similar selective approaches to aid to promote good governance.

Our study had two main limitations. First, the link between democracy and population health is difficult to measure because of the association of democracy with other factors, such as country income or total health expenditure, and the lack of randomised data. We have attempted to control for and minimise the risks of confounding, multicollinearity, and autocorrelation by using a combination of a synthetic control model, fixed-effect regressions, and long-difference regressions, as well as including important covariates. The long-difference model measures long-term trends and removes estimation inefficiency caused by autocorrelation, although this method does not use all of the available data. Fixed-effects regression, alternatively, is susceptible to statistical inefficiency due to autocorrelation, although it uses all available data. The advantage of the synthetic control method is that it allows for the presence of unobserved confounders that vary or are constant in time. However, with observational data such as those at our disposal, it remains impossible to rule out all other potential confounders. Still, we believe that these varied analyses provide evidence of a significant and clear association between democratic experience and health. These results provide a general, global picture of the relationship between democracy and population health; policy makers and practitioners should consider this national-level evidence and other context-specific information in developing health and governance promotion programmes. The factors included in our decomposition analysis together explained less than three-quarters of the total variance for some causes: other factors not considered here might play important roles in dictating those health outcomes, and warrant further research.

Second, although GBD 2016 provides the only comprehensive data on cause-specific mortality and burden in all countries, this effort relies on modelling estimates when data are sparse. Data are more likely to be sparse in low-income countries, particularly for causes like cardiovascular diseases and other non-communicable diseases. The GBD collaboration takes great measures to correct for this bias, both using statistical methods to capture uncertainty and consulting more than 2500 collaborators in 133 countries. However, these data do not directly derive from survey data and thus must be interpreted as modelled estimates. The V-Dem project relies on the responses of multiple country experts to a range of precise survey questions to construct estimates of regime characteristics. The nature of the indicators means that measurement error will occur if experts make a mistake, are subject to bias, or use different rating thresholds. The compilers of the V-Dem data take a number of steps to correct for those potential sources of error, including Bayesian item response modelling.^66^

The results of this study suggest that elections and the health of the people are increasingly inseparable. Democratic institutions and processes, and particularly free and fair elections, can be an important catalyst for improving population health, with the largest health gains possible for cardiovascular and other non-communicable diseases. Conversely, efforts to separate population health from elections and the other hallmarks of democracy might be less successful, especially as aid budgets are stagnant and countries' needs shift to non-communicable diseases, injuries, and adult health. This study suggests that democratic governance and its promotion, along with other government accountability measures, might further enhance efforts to improve population health.

This conclusion might discomfort some. Many global health practitioners might fear that the more political global health assistance becomes, the more it will undermine the productive relationships with local governments on which that assistance depends, spurring suspicions about purportedly well-intentioned outside initiatives. Global health is marketed as a rational, scientific domain with universal aims—the promotion of wellbeing, equity, and reduced poverty. Global health has definite goals and measurable indicators. Politics is a subjective, normative enterprise, on which the signs of progress might be hard to agree. With the political turmoil in the USA and Europe, the case for democracy has never seemed dimmer. Many in global health might see working with authoritarian governments as a preferred, more effective option.

This reticence about democracy promotion is understandable, but it ignores the inevitably political nature of many current global health objectives. As shown by the debates over quality universal health care in the USA, sugar-sweetened beverage taxes in Mexico, or accessibility of affordable cancer drugs in India, many of the issues that are likely to dominate global health in the future are often divisive. These issues involve fundamental questions regarding the role of the state in society and the balance between individual, collective, and commercial interests. Ignoring the role of civil society, a free media, and open and accountable government in resolving these debates undermines efforts to build institutional capacity and the popular support needed for sustained population health improvements. Pretending otherwise is akin to believing that the solution to a nation's crumbling roads and infrastructure is just a technical schematic and cheaper materials.
