# Supplementary material for: The relationships between democratic experience, adult health, and cause-specific mortality in 170 countries between 1980 and 2016: an observational analysis
Source: Lancet. 2019 Apr 20;393(10181):1628–40. doi: 10.1016/S0140-6736(19)30235-1 (PMC6484695; doi:10.1016/S0140-6736(19)30235-1)
Supplement: Supplementary appendix [file mmc1.pdf]

THE LANCET

Supplementary appendix

This appendix formed part of the original submission and has been peer reviewed. We post it as supplied by the authors.

Supplement to: Bollyky TJ, Templin T, Cohen M, Schoder D, Dieleman JL, Wigley S. The relationships between democratic experience, adult health, and cause-specific mortality in 170 countries between 1980 and 2016: an observational analysis. *Lancet* 2019; published online March 13. http://dx.doi.org/10.1016/S0140-6736(19)30235-1.

SUPPLEMENTARY METHODS ANNEX

The relationships between democratic experience, adult health, and cause-specific mortality in 170 countries between 1980 and 2016: an observational analysis

Version: March 1, 2019

SUPPLEMENTARY METHODS ANNEX

1.0 Data

1.1 Introduction

1.2 Data Sources

1.3 Descriptive Statistics

2.0 Statistical Analysis

2.1 Synthetic Control Method

2.2 Shapely Variance Decomposition

2.3 Structural Equation Model

2.4 Leave-one-out Regression

3.0 Tables

1.0 DATA

Part 1.1: Introduction
The objective of this research is to assess whether a country's democratic experience is likely to matter more for health outcomes as the burden of disease shifts to non-communicable diseases (NCDs). We characterize the effect of having democratic experience on a country's health burden, focusing on how and why democracy affects NCDs. Our primary indicators come from several sources: measures of burden by cause from the Global Burden of Disease project, democratic experience indicators from the Varieties of Democracy project, and estimates of country-level health expenditure from the Financing Global Health database. We supplement these with covariates from the Global Burden of Disease project, including data on socio-demographics, disease risk factors, and health-care access and quality.

Using these data, we perform four analyses. The first utilizes the synthetic control method to characterize the effect of democratic transitions on HIV-free life expectancy at fifteen. The second is a Shapley variance decomposition of cause-specific communicable, non-communicable, and injury age-standardized death rates. The third is a structural equation model of the pathways by which democratic experience may improve health. The fourth is a "leave-one-out" regression that systematically drops components of the democratic experience indicator to determine which is most important in population health improvements.

The purpose of this appendix is to provide technical details on our data sources and methodology. We include information on all data sources in Section 1, statistical analyses in Section 2, and relevant country groupings in Section 3.

Part 1.2: Data Sources
We used data from 10 sources to assess the effect of governance on health outcomes, especially non-communicable diseases.

Institute for Health Metrics and Evaluation Global Burden of Disease Study 2016
Death and disability-adjusted life year (DALY) estimates are from the Global Burden of Disease 2016 study.[1] The GBD 2016 reports age- and sex-specific DALY estimates for 333 diseases in 195 countries and territories from 1990 to 2016. The GBD 2016 also reports mortality estimates for 264 causes in 195 countries and territories from 1980 to 2016 and life expectancy from 1970-2016, both by age and sex.[2]

We extract cause-specific age- and sex-standardized death rates at the Level Two of the GBD cause hierarchy (see table 3.4). One way in which we deviate from the GBD cause hierarchy is while IHME combines HIV/AIDS and Tuberculosis as a single Level Two cause, for our analysis, we separate HIV/AIDS and Tuberculosis into individual causes. The rationale behind this is because of differences in treatment amenability, prevention, and prevalence. The other twenty Level Two causes are: diarrhea, lower respiratory, and other common infectious diseases; neglected tropical diseases and malaria; maternal disorders; neonatal disorders; nutritional deficiencies; other communicable, maternal, neonatal, and nutritional diseases; neoplasms; cardiovascular diseases; chronic respiratory diseases; cirrhosis and other chronic liver diseases; digestive diseases; neurological disorders; mental disorders; diabetes, urogenital, blood, and endocrine diseases; musculoskeletal disorders; other non-communicable diseases; transport injuries; unintentional injuries; self-harm and interpersonal violence; and forces of nature, conflict and terrorism, and executions and police conflict.

Additionally, we extract HIV-free life expectancy at fifteen, which provides an estimation of what life expectancy in each country and year would have been had the HIV pandemic had not occurred. The HIV-free life expectancy estimates also did not include mortality shocks caused by war and natural disasters.

Institute for Health Metrics and Evaluation's GBD 2016 Covariates Database
The Institute for Health Metrics and Evaluation's GBD 2016 Covariates database provides access to the 565 covariates used in the GBD 2016 modeling process for 195 countries and territories from 1980 to 2016.[3] We extracted mean years of education, age-standardized prevalence of smoking, prevalence of water sanitation, outdoor

air pollution, indoor air pollution, prevalence of skilled birth attendance, age-standardized obesity rates, mean sodium ingestion, age-standardized HIV prevalence, and mortality shocks. Mortality shocks are the mean mortality rate in the previous ten years due to military operations and terrorism, disasters, and famines (protein energy malnutrition deaths).

Table 1.2.1: GBD Covariates Used

GBD Covariate	Description[3]
Mean years of education	Years of education per capita
Age-standardized prevalence of smoking	The proportion between 0 and 1 of age-standardized smoking prevalence, both sexes combined
Prevalence of water sanitation	Percent of population with access to improved toilet types as defined by the Joint Monitoring Program
Outdoor air pollution	Population weighted average of particulate matter 2.5 micrograms per cubic meter
Indoor air pollution	Household prevalence of cooking with coal or biomass
Prevalence of skilled birth attendance	Percent of women giving birth with a skilled birth attendant (mainly nurses, doctors, and midwives)
Age-standardized obesity rates	Age-standardized prevalence of obesity
Mean sodium ingestion	Grams per day of urinary sodium[4]
Age-standardized HIV prevalence	"HIV age-standardized prevalence"
Mortality shocks	Mortality rate per person due to military operations and terrorism, disasters, and famines (protein energy malnutrition deaths)
Average daily calories intake	Average daily calorie intake (total kcal/p/day avail). We use this as a predictor variable in the synthetic control analysis.

Institute for Health Metrics and Evaluation's Financing Global Health 2017 report

We extract data from two related data sets in IHME's Financing Global Health series. The Development Assistance for Health 2017 database includes health focus area and recipient country-specific development for health estimates for 188 countries from 1990 to 2016 and preliminary estimates for 2017.[5] The Financing Global Health 2017 database includes total health spending estimates (as well as its disaggregated components described below) for 188 countries from 1995 to 2015.[6,7]

Total health spending is defined by IHME as the sum of five mutually exclusive categories: government health spending, development assistance for health, out-of-pocket health spending, private insurance, and nongovernmental organization spending. IHME distinguishes government health spending in two categories: as agent and as source. Government health spending as source is country government spending on health exclusive of development assistance. Government health expenditure as agent measures funds that the country government spends on health, but those funds may be financed from an external source, e.g. a multilateral agency. In our analysis, we utilize development assistance for health and government health spending as source.

Institute for Health Metrics and Evaluation GBD 2016 Population Estimates 1950-2016

The Institute for Health Metrics and Evaluation GBD 2016 Population Estimates provide population estimates by location, age, and sex for 1950-2016.[8] These estimates are comprised of data from two sources: World Population Prospects: 2015 Revision, from the United Nations Population Division and the WHO Human Mortality Database. We use the population growth rate annual percentage, calculated using the exponential growth formula, as a predictor variable for the synthetic control analysis. The growth rate was calculated as follows: $r = \ln(pn/p0)/n$, where r is the exponential rate of growth, ln() is the natural logarithm, pn is the end period population, p0 is the beginning period population, and n is the number of years in between.

Institute for Health Metrics and Evaluation GBD 2016 Socio-demographic Index

The Institute for Health Metrics and Evaluation GBD 2016 Socio-demographic (SDI) index provides SDI estimates for 195 countries and territories from 1970-2016.[9] IHME considers average income per person, educational attainment, and total fertility rate (TFR) to calculate SDI values. In the index, a zero represents the lowest income

per capita, lowest educational attainment, and highest TFR, and one represents the highest income per capita, highest educational attainment, and lowest TFR observed across all geographies.

Institute for Health Metrics and Evaluation Global Educational Attainment 1970-2015
The Institute for Health Metrics and Evaluation provides estimates of average years of educational attainment per capita for people over the age of 15 for the years 1970-2015 by year, sex, and age group for 188 countries, 21 GBD regions, 7 GBD super regions, and the global aggregate.[10] For females 15-44 and for both sexes in the 25 and older age group, the dataset includes age-standardized and population-weighted estimates. We use female educational attainment for ages 15-24 and male educational attainment for ages 15-24 as predictor variables in the synthetic control analysis.

Institute for Health Metrics and Evaluation Infant and Child Mortality Estimates by Country 1970-2010
The Institute for Health Metrics and Evaluation provides estimates of neonatal, postneonatal, childhood, and under-5 mortality for 187 countries between 1970 and 2010.[11] We use under-5 deaths per 1000 live births as a predictor variable in the synthetic control analysis.

Varieties of Democracy
Our indicator for democracy is drawn from the Varieties of Democracy (V-Dem) data set (Version 8).[12] That data set provides over 450 indicators of regime characteristics for 201 countries in the world from 1789 to 2017. Each of the political indicators in that data set is based on the responses of multiple country experts to a range of highly specific survey questions. The ratings of those experts were aggregated using Bayesian item response modelling techniques. This enabled the compilers to correct for measurement error and to create interval-level estimates for each indicator.[13] A significant advantage of the V-Dem data set is that it avoids many of the methodological problems that afflict the democracy indices that are by now standard in the literature (including Polity IV and Freedom House). Those problems include lack of precision, the use of different data sources over time, imprecise coding criteria, and the absence of inter-coder reliability tests.[14,15]

Our democratic stock variable is based on V-Dem's Multiplicative Polyarchy Index (v2x_mpi). That index is the product of the five core components of electoral democracy: suffrage (v2x_suffr), free and fair elections (v2xel_frefair), elected officials (v2x_elecoff), freedom of civil and political organization, including opposition political parties (v2x_frassoc_thick), and freedom of expression, including access to alternative information sources (v2x_freexp_altinf). Those components represent the mechanisms required to achieve responsiveness and accountability between leaders and citizens.[16] The five components are multiplied so as to capture the intuition that each is a necessary and complementary ingredient of democratic rule. Suffrage is not relevant if the executive is not elected, or opposition parties are illegal. Equally, the presence of opposition parties is not relevant if the incumbent can manipulate the electoral results to ensure victory, and so on.[17]

We constructed a stock measure of democracy by taking the sum of each country's score from 1900 to the observation year, applying a 1% annual depreciation rate. Slovakia is missing data in recent years, and so it is not included in our stock variable. This measure reflects both the age and the quality of democratic institutions in each country. As a result it allows us to examine the proximal and distal effects of democratic governance.[18] The policy shifts produced by democratic reform will often require an extended period of time to have an impact on population health. This is especially the case for the prevention and treatment of non-communicable diseases (e.g. anti-tobacco policies, training of health practitioners, investment in infrastructure, etc.). A modest 1% depreciation rate was included because the policies implemented even further back in time (e.g. during the first quarter of the 20th century) will have an increasingly smaller impact on population health in the present. We also use the Varieties of Democracy measure of domestic autonomy as a predictor variable in the synthetic control analysis.

Figure 1.2.1: Map of Democratic Stock, 2016

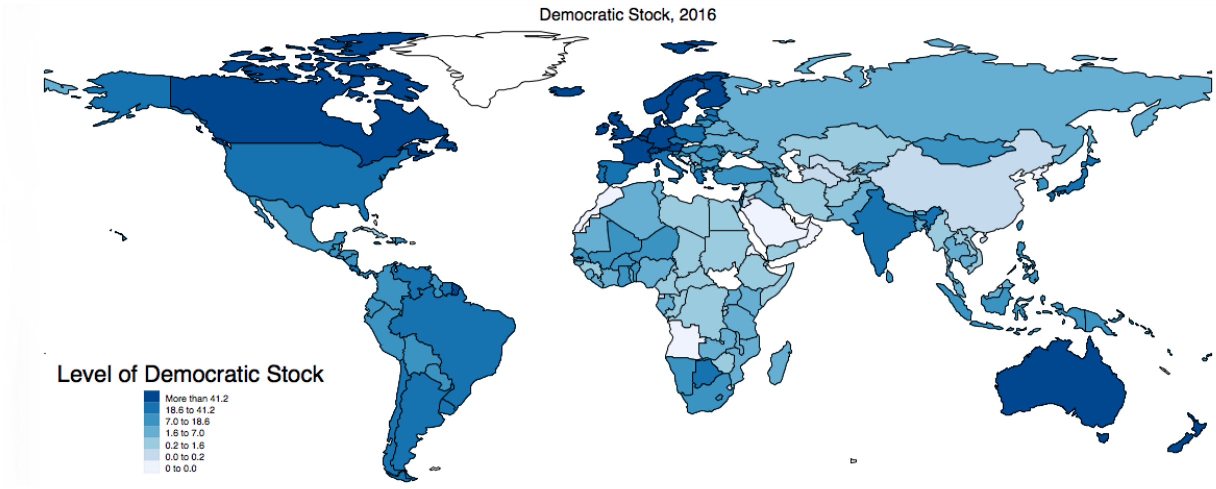

Figure 1.2.2: Map of Changes in Democratic Stock, 1990-2016

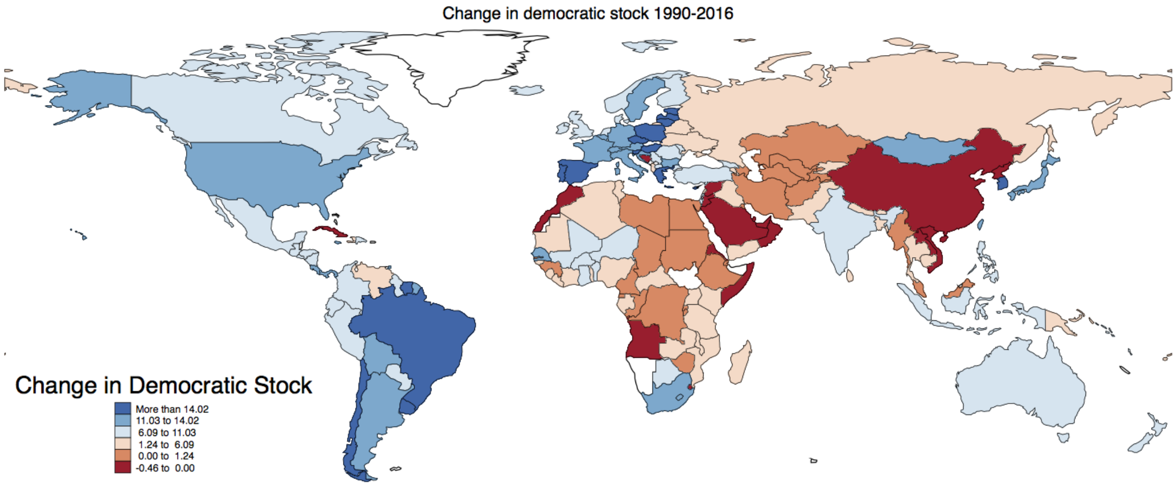

Figure 1.2.3: Map of Percent Changes in Democratic Stock, 1990-2016

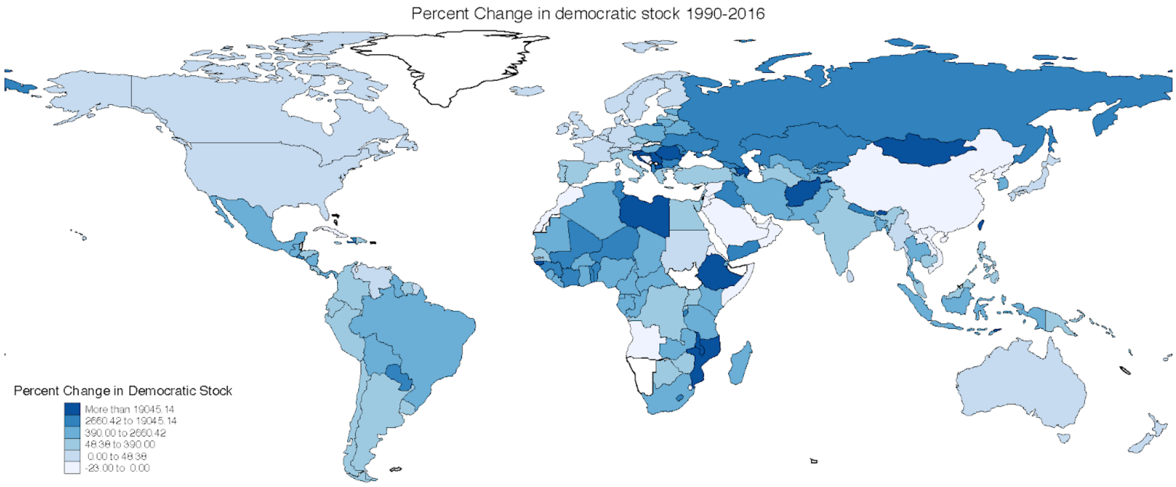

Table 1.2.2: Descriptive Statistics on Distribution of Democratic Stock

Variable	Mean	Standard Deviation	Median (Interquartile Range)	Minimum	Maximum
Democratic Stock (1995)	7.40	11.90	1.42 [7.76]	0.00	46.30
Democratic Stock (2015)	12.55	14.31	6.81 [16.47]	0.00	53.60
Percent change in democratic stock (1995 - 2015)	808.79	4277.26	164.11 [404.39]	-18.21	51359.96
Absolute change in democratic stock (1995 - 2015)	5.14	4.20	4.91 [7.63]	-0.38	13.84

World Development Indicators 2017

Our indicator for urban population comes from the World Development Indicator Urban population (% of total), and it is available from 1960 to 2017 for 189 World Bank member countries, plus 28 other economies with populations of more than 30,000.[19,20] The urban population is defined by national statistical offices, and the indicator is calculated using World Bank population estimates and urban ratios from the United Nations World Urbanization Prospects.

Data from 'Ruggedness: The blessing of bad geography in Africa' (Nunn and Puga 2012)

We use the percentage of land area with a tropical climate and the average distance to the nearest ice-free coast from "Ruggedness: The blessing of bad geography in Africa" by Nathan Nunn and Diego Puga (2012) as predictor variables in the synthetic control analysis.[21] The percentage of land area with a tropical environment is determined by classifying each cell on a 30 arc-minute grid covering the land area of the world into one of 31 climates in the Köppen-Geiger climate classification. The authors use this data to calculate the percentage of the land surface area of each country that has any of the four Köppen-Geiger tropical climates. For the distance to the nearest ice-free coast, the authors compute the distance to the nearest ice-free coast in thousands of kilometers for every point in each country using an equi-rectangular projection with standard parallels at 30 degrees. They then averaged this distance across all land in each country not covered by inland water features.

Part 1.3: Descriptive Statistics

Table 1.3.1: Descriptive Statistics of Variables Included in All Analyses

Variable Name		Mean	Std. Dev	Min.	Max.		Observations	Years
Multiplicative polyarchy index (stock)	overall	9.49	13.06	0	53.9	N	4563	1990-2016
	between		12.84	0	49.24	n	169	
	within		2.58	-0.11	18.2	T	27	
Multiplicative polyarchy index (ordinal) [subset for Analysis 1]	overall	0.26	0.40	0	1	N	1820	1990-2016
	between		0.37	0	1	n	70	
	within		0.16	-0.70	0.94	T	26	
MPI without free association	overall	11.82	14.9	0	59.63	N	4583	1990-2016
	between		14.63	0	54.65	n	170	
	within		2.99	1.42	21.64	T	26.9588	
MPI without free & fair	overall	13.01	14.31	0	57.87	N	4583	1990-2016
	between		13.93	0	53.67	n	170	
	within		3.38	2.98	22.27	T	26.9588	
MPI without suffrage	overall	10.27	14.06	0	57.09	N	4583	1990-2016
	between		13.85	0	52.73	n	170	
	within		2.61	0.64	19.15	T	26.9588	
MPI without elected officials	overall	10.3	13.59	0	55.17	N	4583	1990-2016
	between		13.36	0	50.54	n	170	
	within		2.65	0.77	19.05	T	26.9588	
MPI without free expression	overall	11.58	14.42	0	58.27	N	4583	1990-2016
	between		14.15	0	54.11	n	170	
	within		2.93	1.6	20.96	T	26.9588	
Vote buying	overall	19.34	16.1	0	65.22	N	4583	1990-2016
	between		15.95	0	60.47	n	170	
	within		2.45	9.02	29.23	T	26.9588	
Electoral board autonomy	overall	22.28	17.07	0	66.83	N	4583	1990-2016
	between		16.73	0	62.64	n	170	
	within		3.58	11.66	32.01	T	26.9588	

Electoral board capacity	overall	22.79	17.47	0	67.19	N	4583	1990-2016
	between		17.18	0	62.81	n	170	
	within		3.41	12.06	33	T	26.9588	
Election voter registry	overall	18.27	16.01	0	62.5	N	4583	1990-2016
	between		15.75	0	57.66	n	170	
	within		3.1	7.47	28.35	T	26.9588	
Other voting irregularities	overall	18.96	16.03	0	63.95	N	4583	1990-2016
	between		15.83	0	59.27	n	170	
	within		2.72	8.05	28.64	T	26.9588	
Election government intimidation	overall	18.17	15.57	0	62.67	N	4583	1990-2016
	between		15.31	0	57.55	n	170	
	within		3.04	7.77	28.27	T	26.9588	
Election other electoral violence	overall	18.67	15.13	0	60.52	N	4583	1990-2016
	between		14.89	0	56.13	n	170	
	within		2.9	8.13	28.71	T	26.9588	
HIV-free Life Expectancy at 15	overall	58.16	5.15	44.63	69.23	N	4590	1990-2015
	between		4.91	45.42	66.99	n	170	
	within		1.59	50.61	64.24	T	27	
Urbanicity	overall	0.53	0.23	0.05	1	N	4590	1990-2016
	between		0.23	0.09	1	n	170	
	within		0.04	0.39	0.71	T	26.9704	
Sodium	overall	4	1.27	1.37	10.25	N	4590	1990-2016
	between		1.28	1.37	10.23	n	170	
	within		0.01	3.9	4.22	T	27	
Obesity Prevalence	overall	0.14	0.09	0	0.49	N	4590	1990-2016
	between		0.08	0.01	0.48	n	170	
	within		0.03	0.03	0.23	T	27	
Indoor Air Pollution	overall	0.41	0.38	0	1	N	4590	1990-2016
	between		0.38	0	1	n	170	
	within		0.07	0.13	0.85	T	27	
Outdoor Air Pollution	overall	17.62	10.81	2.94	75.54	N	4590	1990-2016

	between		10.57	3.38	70.25	n	170	
	within		2.44	2.67	48.84	T	27	
Smoking Prevalence	overall	0.16	0.08	0.01	0.36	N	4590	1990-2016
	between		0.08	0.01	0.34	n	170	
	within		0.02	0.07	0.26	T	27	
Access to Safe Water	overall	0.66	0.33	0.01	1	N	4590	1990-2016
	between		0.33	0.05	1	n	170	
	within		0.08	0.21	1.12	T	27	
Mean Years of Education (Both sexes)	overall	7.71	3.63	0.69	15.18	N	4590	1990-2016
	between		3.55	1.3	14.36	n	170	
	within		0.82	5.26	10.66	T	27	
Skilled Birth Attendance	overall	0.81	0.23	0.09	1	N	4590	1990-2016
	between		0.22	0.16	0.99	n	170	
	within		0.07	0.48	1.2	T	27	
GDP per capita	overall	13694.6	16659	294.31	125411.7	N	4590	1990-2016
	between		16107.21	513.26	91443.49	n	170	
	within		4421.55	-23701.67	47662.78	T	27	
Mortality Rate Shock	overall	-4.97	5.78	-15.64	0	N	4590	1990-2016
	between		3.75	-12.48	0	n	170	
	within		4.41	-18.18	7.51	T	27	
Maternal Education	overall	8.28	3.93	0.56	15.48	N	4590	1990-2016
	between		3.82	1.18	14.62	n	170	
	within		0.97	4.81	12.43	T	27	
Age-standardized Cardiovascular Disease Death Rate	overall	384.64	161.13	94.69	964.44	N	4590	1990-2016
	between		152.12	140.17	910.61	n	170	
	within		54.35	205.31	590.67	T	27	
Age-standardized Cirrhosis Death Rate	overall	26.3	17.71	3.42	145.75	N	4590	1990-2016
	between		17.36	3.8	131.95	n	170	
	within		3.73	5.84	46.27	T	27	
Age-standardized Diabetes Death Rate	overall	71.21	50.16	10.93	468.39	N	4590	1990-2016
	between		49.06	14	425.58	n	170	
	within		11.08	-40.07	138.44	T	27	

Age-standardized Diarrheal Disease Death Rate	overall	139.99	153.3	5.2	701.29	N	4590	1990-2016
	between		146.81	8	520.67	n	170	
	within		45.51	-72.46	389.39	T	27	
Age-standardized Digestive Disorders Death Rate	overall	26.97	17.69	4.74	92.96	N	4590	1990-2016
	between		17.17	5.51	82.14	n	170	
	within		4.45	4.14	62.46	T	27	
Age-standardized Unintentional Injuries Death Rate	overall	33.16	18.04	4.4	94.46	N	4590	1990-2016
	between		17.31	6.35	79.86	n	170	
	within		5.24	10.56	65.03	T	27	
Age-standardized Forces Injuries Death Rate	overall	6.14	127.8	0.01	8125.12	N	4590	1990-2016
	between		26.24	0.01	314.25	n	170	
	within		125.09	-308.04	7817.02	T	27	
Age-standardized HIV Death Rate	overall	57.3	162.17	0	1460	N	4590	1990-2016
	between		142.4	0.03	823.64	n	170	
	within		78.33	-683.64	762.59	T	27	
Age-standardized Maternal Causes Death Rate	overall	4.55	5.08	0.32	43.86	N	4590	1990-2016
	between		4.93	0.33	33.8	n	170	
	within		1.29	-7.24	16.15	T	27	
Age-standardized Neonatal Causes Death Rate	overall	1.32	0.76	0.16	4.13	N	4590	1990-2016
	between		0.73	0.19	3.7	n	170	
	within		0.2	0.37	3.46	T	27	
Age-standardized Neoplasms Death Rate	overall	139.96	35.21	62.83	296.83	N	4590	1990-2016
	between		33.93	65.73	283.79	n	170	
	within		9.76	89.98	180.98	T	27	
Age-standardized Neurological Causes Death Rate	overall	45.94	9.13	27.35	91.44	N	4590	1990-2016
	between		9.05	29.29	88.57	n	170	
	within		1.36	35.37	53.07	T	27	
Age-standardized NTDs and Malaria Death Rate	overall	22.91	47.89	0.01	479.04	N	4590	1990-2016
	between		46.06	0.01	213.9	n	170	
	within		13.57	-93.49	288.06	T	27	

Age-standardized Nutritional Disorders Death Rate	overall	14.72	27.6	0.03	428.39	N	4590	1990-2016
	between		22.56	0.06	124.12	n	170	
	within		15.99	-107.05	318.99	T	27	
Age-standardized Other Group 1 Causes Death Rate	overall	6.35	7.35	0.24	42.4	N	4590	1990-2016
	between		7.05	0.31	31.82	n	170	
	within		2.17	-4.3	26.57	T	27	
Age-standardized Other NCDs Causes Death Rate	overall	12.14	6.3	2.39	47.3	N	4590	1990-2016
	between		5.9	3.79	36.02	n	170	
	within		2.24	1.23	41.13	T	27	
Age-standardized Respiratory Causes Death Rate	overall	55.16	43.92	9.69	433.15	N	4590	1990-2016
	between		42.57	14.2	384.98	n	170	
	within		11.29	-30.74	141.21	T	27	
Age-standardized Tuberculosis Death Rate	overall	46.28	75.2	0.19	532.95	N	4590	1990-2016
	between		70.49	0.33	502.94	n	170	
	within		26.74	-120.48	329.61	T	27	
Age-standardized Transport Death Rate	overall	23.04	12.02	3.83	83.74	N	4590	1990-2016
	between		11.29	6.31	65.28	n	170	
	within		4.2	3.31	75.61	T	27	
Age-standardized Violence Death Rate	overall	19.34	12.9	3.06	81.21	N	4590	1990-2016
	between		12.43	3.5	69.15	n	170	
	within		3.55	-1.3	42.28	T	27	
Tropical Climate	overall	37.04	44.15	0	100	N	4563	1990-2016
	between		44.27	0	100	n	170	
	within		0	37.04	37.04	T	27	
Distance to Coast	overall	0.34	0.41	0	2.21	N	4563	1990-2016
	between		0.42	0	2.21	n	170	
	within		0	0.34	0.34	T	27	
DAH per capita	overall	14.16	25.86	0	446	N	3570	1995-2016
	between		18.09	-0.19	94.57	n	170	
	within		18.53	-76.37	369.54	T	21	
Total Health Spending per capita	overall	1056.09	1410.07	16	9839	N	3570	1995-2016

	between		1373.98	27.9	7710.19	n	170	
	within		333.22	-1539.01	3184.9	T	21	
Government Health Spending as Source per capita	overall	702.82	1063.43	1	5992	N	3570	1995-2016
	between		1033.61	2.67	4787.48	n	170	
	within		261.76	-1381.66	2261.91	T	21	
Prepaid Private Health Spending per capita	overall	86.78	263.41	0	3780	N	3570	1995-2016
	between		258.48	0	3058.91	n	170	
	within		54.28	-854.13	807.87	T	21	
Out-of-pocket Health Spending per capita	overall	252.34	256.99	3	1714	N	3570	1995-2016
	between		245.15	4.76	1399.05	n	170	
	within		79.27	-150.28	802.44	T	21	

Analysis One Subset

HIV-free Life Expectancy at 15	overall	55.55	4.59	43.49	68.92	N	3220	1970-2015
	between		4.12	46.74	62.82	n	70	
	within		2.08	47.72	65.12	T	46	
GDP per capita	overall	9333.40	15771.7	372.30	130530	N	3220	1970-2015
	between		14789.7	597.32	95322	n	70	
	within		5750.46	-31941.4	49254.3	T	46	
Female Education (ages 15-24)	overall	6.05	3.24	0.48	14.48	N	3220	1970-2015
	between		2.77	1.12	11.16	n	70	
	within		1.71	1.63	10.63	T	46	
Male Education (ages 15-24)	overall	6.50	2.75	1.15	13.94	N	3220	1970-2015
	between		2.33	2.23	11.16	n	70	
	within		1.50	2.55	10.31	T	46	
Under-5 Mortality	overall	101.07	76.75	2.5	370.3	N	2867	1970-2015
	between		68.64	9.90	257.79	n	70	
	within		35.31	-11.14	250.28	T	40.96	
Urbanicity	overall	45.86	22.95	3.20	100	N	3220	1970-2015
	between		21.68	11.54	100	n	70	
	within		7.95	9.83	71.45	T	46	

Population Growth	overall	11.58	14.42	0	58.27	N	3220	1970-2015
	between		14.15	0	54.11	n	70	
	within		2.93	1.6	20.96	T	46	
Tropical	overall	40.21	43.33	0	100	N	3220	1970-2015
	between		43.64	0	100	n	70	
	within		0	40.21	40.21	T	46	
Distance to coast	overall	0.36	0.39	0	1.84	N	3220	1970-2015
	between		0.39	0	1.84	n	70	
	within		0	0.36	0.36	T	46	
Kilocalories	overall	2486.7	506.52	1367.53	4114.04	N	2520	1970-2015
	between		471.70	1568.71	3891.4	n	70	
	within		192.77	1501.73	3299.66	T	36	
Domestic Autonomy	overall	0.93	0.80	-3.18	2.20	N	3220	1970-2015
	between		0.58	-0.55	2.05	n	70	
	within		0.56	-3.07	2.43	T	46	
Ordinal MPI	overall	0.16	0.33	0	1	N	3217	1970-2015
	between		0.24	0	0.90	n	70	
	within		0.24	-0.45	0.98	T	45.96	

2.0 STATISTICAL ANALYSIS

Part 2.1: Synthetic Control Method
The synthetic control method estimates the impact of an event on an outcome by constructing a "synthetic control" of weighted comparator countries. In our analysis, the event was democratic transition, the outcome was HIV-free life expectancy at 15, and the synthetic control reflects countries that did not undergo a democratic transition. Thus, the synthetic control estimates the life expectancy trajectory that each transitioning country would have taken in the absence of the transition. The synthetic control method was first used by Abadie and Gardeazabal to show the effect of conflict on economic growth and subsequently the effect of an anti-tobacco program on cigarette sales.[22,23] This approach is particularly useful when no single untreated unit exists that is sufficiently similar to the treated unit. This is often the case for studies based on large aggregate units such as countries or regions.

The method was completed in three steps. First, we identified 15 countries that experienced an unambiguous democratic transition from 1980 to 2000. Second, we created synthetic controls for each of those 15 countries. The synthetic controls are a weighted set of countries that did not experience a democratic transition from 1970 to 2015. Weights were set such that the synthetic control compares directly to corresponding treatment countries closely on the pre-treatment characteristics described below. Third, the effect of democratisation was estimated by comparing the life expectancy trajectories of the democratising countries and their synthetic controls. Effect significance was determined by a permutation test.

Identification of transitioning and non-transitioning states

Countries were classified as at least "minimally democratic" if they received a score of 1 on the ordinal version of V-Dem's Multiplicative Polyarchy Index (e_v2x_mpi_3C). Only those countries that became democratic between 1980 and 2000, and that stayed entirely democratic until at least 2015, were retained for the analysis. This meant that countries that became democratic before 1980 or after 2000 were excluded from the analysis. This provided us with at least 10 years lag time to construct the synthetic control and at least 15 years lead time to observe the effect. Based on those criteria we identified 15 "treatment" countries. In addition, we identified 55 "donor" countries that did not transition from autocracy to democracy at any time between 1970 and 2015. The treatment and donor countries are listed in Table 2.1.1.

Note: Some would classify Botswana and South Africa as non-democratic because there has not been a change in government in those countries since the transition took place.[24] Excluding those two countries from the analysis did not change the overall observed effect, but it did improve the statistical significance of the results.

Table 2.1.1 Countries Used in the Synthetic Control Analysis

Country	Governance Status
Afghanistan	Autocracy
Albania	Autocracy
Algeria	Autocracy
Angola	Autocracy
Bolivia	Democratic Transition: 1990
Botswana	Democratic Transition: 1990
Brazil	Democratic Transition: 1988
Bulgaria	Democratic Transition: 1991
Burkina Faso	Autocracy
Cambodia	Autocracy
Cameroon	Autocracy
Cape Verde	Democratic Transition: 1992
Chad	Autocracy
China	Autocracy
Cote d'Ivoire	Autocracy
Cuba	Autocracy
Cyprus	Democratic Transition: 1981
Democratic Republic of the Congo	Autocracy

Djibouti	Autocracy
Egypt	Autocracy
El Salvador	Autocracy
Equatorial Guinea	Autocracy
Ethiopia	Autocracy
Gabon	Autocracy
Guinea	Autocracy
Guyana	Autocracy
Haiti	Autocracy
Honduras	Autocracy
Hungary	Democratic Transition: 1990
Iran	Autocracy
Iraq	Autocracy
Jordan	Autocracy
Kenya	Autocracy
Kuwait	Autocracy
Laos	Autocracy
Lesotho	Autocracy
Libya	Autocracy
Malawi	Autocracy
Malaysia	Autocracy
Mali	Autocracy
Mauritania	Autocracy
Mexico	Democratic Transition: 2000
Mongolia	Democratic Transition: 1992
Morocco	Autocracy
Mozambique	Autocracy
Myanmar	Autocracy

North Korea	Autocracy
Oman	Autocracy
Panama	Democratic Transition: 1991
Philippines	Autocracy
Poland	Democratic Transition: 1990
Qatar	Autocracy
Russia	Autocracy
Rwanda	Autocracy
Saudi Arabia	Autocracy
Senegal	Democratic Transition: 1996
Seychelles	Autocracy
Sierra Leone	Autocracy
Singapore	Autocracy
Somalia	Autocracy
South Africa	Democratic Transition: 1999
South Korea	Democratic Transition: 1989
Swaziland	Autocracy
Syria	Autocracy
Taiwan	Democratic Transition: 1999
Tanzania	Autocracy
The Gambia	Autocracy
Togo	Autocracy
Zambia	Autocracy
Zimbabwe	Autocracy

Estimation
In order to maximize the pre-treatment fit of the model we used the following predictors of adult life expectancy. GDP per capita and urbanicity capture economic wealth and development. Female and male educational attainment for ages 15-24 are included because education is a key driver of population health. Population growth rate and under-5 mortality capture the extent to which each country has undergone the demographic transition. Average calorie intake reflects nutrition levels, as well as the distribution of the food supply within each country. The limit to

how much food can be hoarded or consumed means that increases in mean calorie intake typically reflect an increase in the food supply for the poorest, at least in low- and middle-income countries.[25] Percentage of the land area with a tropical climate indicates the likelihood that a population is exposed to vector-borne diseases. Average distance to an ice-free coast is a key geographic determinant of long run economic growth and it may also affect the government's ability to supply public goods. Finally, degree of domestic control over policy is included because external governments may be less responsive to the health needs of the local population.

Table 2.1.2: Predictor Variables in the Synthetic Control Analysis

**Variable**	**Description**	**Source**
Female education	Female educational attainment for ages 15-24	IHME Educational Attainment 1970-2015
Male education	Male educational attainment for ages 15-24	IHME Educational Attainment 1970-2015
Tropical Climate	Percent land area with tropical climate	Nunn and Puga, 2012
Distance to Coast	Average distance to nearest ice-free coast	Nunn and Puga, 2012
Urbanicity	Urban population, percentage of total	WDI*
GDP Per Capita	GDP per capita	IHME
Energy	Average daily calorie intake (total kcal/p/day avail)	GBD 2016 Covariates
Under-5 mortality	Under-5 deaths per 1000 live births	IHME Infant and Child Mortality Estimates by Country 1970-2010
Population growth	Population growth rate, annual percent (calculated using exponential growth formula)	IHME Population Estimates
Domestic Autonomy	A measure of how autonomous from the control of other states a state is with respect to the conduct of domestic policy	Varieties of Democracy (version 8)

*Taiwan is not included in the WDI urbanicity data, so its values come from the Varieties of Democracy urbanization data, which exists through 2010. The data from 2011-2015 is extrapolated.

15 synthetic countries were constructed corresponding to each of the 15 transitioning countries. This was achieved by weighting the continuously autocratic countries based on the degree to which their predictors resemble that of the transitioning country during the pre-treatment period (for a formal presentation of the method see Abadie et al, 2010, p. 493).[23] Table 2.1.3 compares the average pretreatment predictors for the treated countries with those of the synthetic countries, as well as with the average of the 55 untreated countries.

Table 2.1.3: Predictor Variable Balance

Predictor	Treated	Synthetic	Average of 55 Untreated Countries
Female Education	6.65	6.07	4.64
Male Education	6.77	6.50	5.34
Tropical Climate	21.69	29.39	45.26
Distance to Coast	0.36	0.66	0.36
Urbanicity	50.77	53.98	39.93
GDP Per Capita	6816.96	10,137.25	7743.48
Under-5 mortality	69.05	81.57	119.94
Population Growth	1.78	1.91	2.60
Energy	2683.79	2568.57	2953.37
Domestic Autonomy	0.64	0.98	1.27

Note: All variables are the average for the ten years prior to treatment.

Inference

Divergence between each transitioning country and its synthetic control after the transition year reflects the estimated effect of democratisation on adult life expectancy. Degree of fit during the pre-treatment period indicates how likely it is that each synthetic control reliably predicts the counterfactual path of the treated country. However, there remains some uncertainty over the path that the treated country would have taken in the absence of the treatment. To address that concern, we ran ‘placebo’ tests using each of the continuously autocratic countries (see Abadie et al, 2010, p. 496-7).[23] The same model is run on each of the untreated units, assuming they were treated at the same time. If the distribution of placebo effects displays a number of effects as large as the treatment effect, then it is likely that that effect was observed by chance. Thus, our test for statistical significance is the proportion of untreated countries that produced the same or greater estimated effect as the treated country. Note that this inferential method does not produce confidence intervals because the actual treatments were not randomly assigned across countries (see Abadie et al, 2015, p. 500).[26]

The placebo effects may be artificially large if those units were not matched well in the pre-treatment period. To control for this possibility, we excluded from inference those placebos where the pre-treatment match quality (root mean square prediction error) was 1.5 times worse that the treated unit. Nevertheless, that adjustment did not significantly alter the resulting p-values.

Software

The synth_runner package for Stata was used to implement this method.[27] Based on the approach developed by Cavallo and colleagues, this package produces the average effect and p-values for multiple treatment units, with different treatment years.[28]

The following table lists the average effect on HIV-free life expectancy after 5 and 10 years, for each age level. There is a statistically significant effect, after 10 years, across all ages.

Table 2.1.4: Average Effect of Democratic Transitions on HIV-free Life Expectancy Across the Lifespan

HIV-free life expectancy at:	Average effect after 5 years (%)	Average effect after 10 years (%)	Average p value after 10 years
< 1	1.36	1.83	0.0422
1	1.59	1.83	0.0274
5	2.16	2.46	0.0024
10	2.5	2.81	0.0016
15	2.77	3.1	0.0012
20	3.02	3.37	0.0011
25	3.21	3.54	0.0012
30	3.44	3.75	0.0014
35	3.68	3.95	0.0018
40	3.87	4.2	0.002
45	3.96	4.42	0.0019
50	3.97	4.61	0.0024
55	4.05	4.89	0.0027
60	4.01	5.07	0.0028
65	4.18	5.47	0.0017
70	3.73	5.29	0.0036
75	3.59	4.85	0.0055
80	2.8	3.87	0.0169
85	2.46	3.48	0.0248
90	2.08	3.88	0.0105

Table 2.1.5. Actual and Synthetic Life Expectancy at 15 by Relative Year

Year	Actual Life Expectancy	Synthetic Life Expectancy	P-values
-11	0.98212475	0.98256481	
-10	0.98397434	0.98376399	
-9	0.9868148	0.9859013	
-8	0.98859364	0.98832923	
-7	0.98988807	0.98981071	
-6	0.9922573	0.99107444	
-5	0.99428707	0.9942196	
-4	0.99711174	0.99956524	
-3	0.99858701	1.0018227	
-2	0.99929887	1.0014801	
-1	1	1.0001334	
1	1.00129	0.99824494	0.021767
2	1.0006196	0.99519658	0.04879
3	1.0021172	0.98958212	0.005158
4	1.0029415	0.98158598	0.000232
5	1.004455	0.97667706	0.000025
6	1.0060726	0.97847444	0.000036
7	1.0080941	0.98313165	0.000393
8	1.0111431	0.98700225	0.001885
9	1.0144066	0.98802572	0.002448
10	1.0171829	0.98568207	0.001216
11	1.0200438	0.98319978	0.000682
12	1.0234029	0.98313767	0.000676
13	1.0261114	0.98351312	0.000823
14	1.0292029	0.98436987	0.00091
15	1.0331285	0.98694336	0.001098
16	1.0374321	0.9896962	0.001151

Robustness

The outcome variable for the benchmark model is adult life expectancy, exclusive of HIV mortality. This is because the primary focus of this study is non-communicable diseases and because development aid for HIV/AIDS has been substantial, potentially cloaking the policy shortcomings of autocratic regimes. For the sake of robustness we also ran the synthetic control analysis for adult life expectancy that incorporates mortality due to HIV. In order to accurately model HIV-included life expectancy, we narrowed the set of predictors to GDP per capita, female and male educational attainment, tropical climate, distance to nearest ice-free coastline, and domestic autonomy, but added age-standardized HIV prevalence.[3] The results of this robustness check are presented in Figure 2.1.1 A and B. In keeping with our baseline results, we find that democratic transitions are associated with an average improvement in life expectancy at 15 of 2.6% (p-value = 0·026) after 10 years, corresponding to an absolute difference of 0.026.

Figure 2.1.1A: Average Effect of Democratic Transitions on Life Expectancy at 15

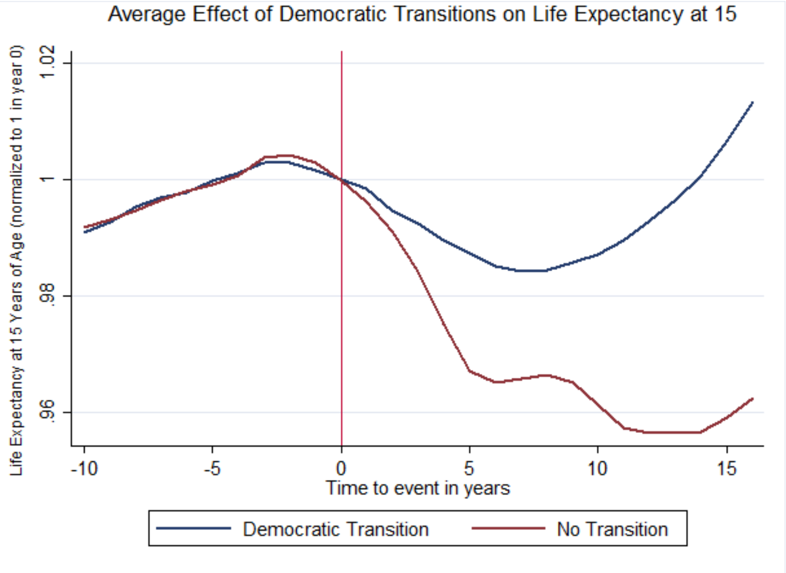

Figure 2.1.1B: Empirical Test for Effect Significance (p-values)

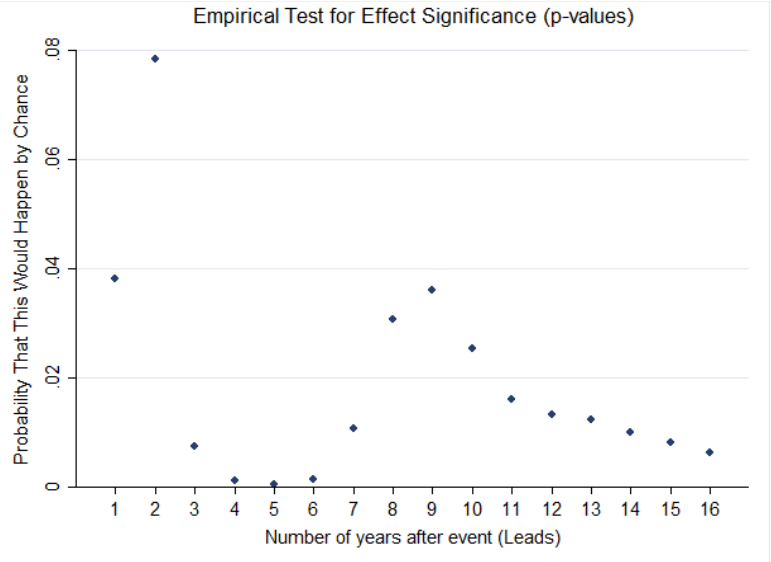

Part 2.2: Shapely Variance Decomposition
We decompose the changes observed in mortality rates predictable from changes in determinants of health in order to understand the heterogeneous effects on cause-specific mortality of these determinants. We use the Shapley variance decomposition method in the relaimpo package (for R version 3.4.3) to quantify the relative importance of each variable.[29]

R^2 as Explained Variance
We use R^2 to quantify explained variance. R^2, or the coefficient of determination, calculates the fraction of variance in the outcome that can be predicted, or "explained" by the independent variables in a regression equation. We calculate R^2 in the usual way:[30]

$$SS_{total} = \sum_i (y_i - \bar{y})^2 \qquad SS_{residual} = \sum_i (y_i - \hat{y}_i)^2 \qquad R^2 = 1 - \frac{SS_{residual}}{SS_{total}}$$

Where there are i observations, ŷ is the predicted value from the regression equation, and ȳ is the average value of the outcome variable. SS_{total} is the total sum of squares, and $SS_{residual}$ is the residual sum of squares. For example, we interpret an R^2 of 0.25 as explaining 25% of the variance in age-standardized mortality, leaving 75% of the changes in mortality unexplained by the predictors in the regression equation.

Regression Equations
We complete two variance decomposition analyses. The first decomposes variance in all GBD Level Two causes, based on a panel of county-year observations. The regression equation is:

$$\begin{aligned} \textit{Death rate}_{it} = {} & \beta_0 + \beta_1 (\textit{Democratic Stock}_{it}) + \beta_2 \, ln(\textit{GDP per capita}_{it}) + \beta_3 \, ln(\textit{urbanicity}_{it}) \\ & + \beta_4 (\textit{Mortality Shock}_{it}) + \beta_5 (\textit{DAH per capita}_{it}) \\ & + \Sigma \, \tau_t 1(\textit{year}) + \Sigma \, \delta_i 1(\textit{iso}) + \epsilon_{it} \end{aligned}$$

The predictor variables are democratic stock, GDP per capita, urbanicity, DAH per capita, mortality shocks, and indicator variables for year. In order to assess within country variation, we de-mean all variables according to country means. The regression equation includes country indicators to express that marginal effects are "within country" effects, but we do not decompose the variance of the country effects.

The second decomposes variance in each year across countries in all GBD Level Two causes. The regression equation estimated for each year and cause is:

$$\begin{aligned} \textit{Death rate}_{i} = {} & \beta_0 + \beta_1 (\textit{Democratic Stock}_{i}) + \beta_2 \, ln(\textit{GDP per capita}_{i}) + \beta_3 \, ln(\textit{urbanicity}_{i}) \\ & + \beta_4 (\textit{Mortality Shock}_{i}) + \beta_5 (\textit{DAH per capita}_{i}) + \epsilon_{i} \end{aligned}$$

The predictor variables are the same as the first decomposition analysis, except there are no country- or year-fixed effects.

For each analysis, we used 170 countries, 1995 through 2016, to estimate the regression equations. We are restricted to starting the analysis at 1995 due to the variable DAH per capita.

Intuition about Variance Decomposition
The decomposition estimates are a function of the estimated marginal effect, the observed variation in the independent variable, and the observed variation in the dependent variable. While the variation of the independent variable is the same across equations, the variation of the dependent variable changes (as well as the estimated marginal effects). An easy way to think about this is to imagine that the estimated marginal effect for two diseases is the same, but if the variation in the dependent variable is much larger for one of the two, then the share of the variation explained by the independent variable will differ. To apply this logic to sample cases, the marginal effect of democracy on HIV mortality is very large, but variation across time and countries in HIV mortality is enormous, so the decomposition shows a share of variation HIV mortality explained by democracy is really small. Alternatively, the marginal effect of democracy on cancer morality is much smaller, but variation across time and countries in

cancer mortality is also relatively small, so the decomposition shows the share of the variation of cancer mortality explained by democracy is actually very large.

Variance Decomposition Formulas

We use the Lindeman, Merenda, and Gold relative importance metric to partition R^2 into the variance explained by each predictor.[31] Similar to Shapely's decomposition metric for allocation marginal product, this algorithm will consider all orderings of the regression list and the marginal contribution to R^2 of each predictor. Specifically, for each ordering of the regression list, the relaimpo package performs stepwise regression, adding each variable one at a time, and calculating the R^2 to attribute a change in R^2 to that variable.[29] The relative importance proportions for each variable are averaged across orderings. Numerically, this algorithm is:

$$LMG(predictor) = \frac{1}{p!} \sum_{orderings} seq\ R^2((predictor) \mid ordering)$$

where $seq\ R(predictor \mid previous\ variables\ in\ ordering) =$

$$R^2(predictor \cup previous\ variables\ in\ ordering) - R^2(previous\ variables\ in\ ordering)$$

where $R^2(x)$ is shorthand to represent the R^2 associated with a regression the outcome using x predictors.[29]

Figure 2 of the article ("Explained Changes in Mortality Due To Democracy and Other Determinants of Health") shows the proportion of variance in Global Burden of Disease Level Two cause-specific age-standardised mortality explained by democracy and other determinants of health. Here, we show below the proportion of variance of each variable included in this analysis.

Table 2.2.1. Explained Changes in Mortality Due to Democracy and Other Determinants of Health: Proportions of Variance for Each Variable in our Analysis

Disease	Year	GDP per capita	Urbanicity	DAH per capita	Mortality Shock	Democracy	Total Explained by Model (R-squared)
Cardiovascular	0.2613	0.1183	0.0413	0.0065	0.0005	0.2227	0.6507
Respiratory	0.2326	0.1879	0.0652	0.0043	0.0011	0.0759	0.5669
Cirrhosis	0.1075	0.0218	0.0698	0.0029	0.0022	0.0614	0.2656
Diabetes	0.0263	0.0071	0.0140	0.0064	0.0019	0.0044	0.0603
Cancer	0.0933	0.0607	0.0169	0.0112	0.0016	0.0950	0.2787
Diarrhea	0.2710	0.2374	0.1284	0.0083	0.0002	0.0959	0.7413
Digestive	0.2487	0.1700	0.1189	0.0059	0.0002	0.0904	0.6341
Forces	0.0123	0.0224	0.0063	0.0026	0.3963	0.0043	0.4442
Maternal	0.2266	0.1774	0.0982	0.0056	0.0004	0.0656	0.5739
Mental	0.0106	0.0258	0.0023	0.0004	0.0001	0.0045	0.0436
Musculoskeletal	0.0041	0.0006	0.0031	0.0004	0.0011	0.0033	0.0125
NTDs + Malaria	0.1429	0.0646	0.1042	0.0082	0.0001	0.0418	0.3618
Neurological	0.0113	0.0024	0.0259	0.0060	0.0001	0.0317	0.0773
Nutrition	0.1779	0.0886	0.0472	0.0047	0.0006	0.0440	0.3630
Other Communicable	0.2188	0.1826	0.0942	0.0060	0.0008	0.0864	0.5888
Other NCDs	0.1576	0.0914	0.0289	0.0025	0.0006	0.1268	0.4079
Violence	0.0975	0.1250	0.0359	0.0072	0.0002	0.0470	0.3127
Transport	0.1745	0.0665	0.0261	0.0045	0.0004	0.1778	0.4499
Falls + Fire	0.2791	0.1881	0.0722	0.0050	0.0003	0.1221	0.6669
TB	0.2725	0.1989	0.0835	0.0091	0.0011	0.1653	0.7304
HIV	0.0691	0.0097	0.0045	0.0004	0.0003	0.0283	0.1124

Table 2.2.2. Explained Changes in Mortality Due to Democracy Over Time: Proportions of Variance for Democracy, 1995 and 2015

Disease	Total R^2, 1995	Total R^2, 2015	Democracy R^2, 1995	Democracy R^2, 2015	Share of R^2 Attributable to Democracy, 1995	Share of R^2 Attributable to Democracy, 2015
Cardiovascular	0.2265	0.5048	0.1443	0.2523	0.6373	0.4997
Respiratory	0.4075	0.4347	0.0472	0.0316	0.1159	0.0728
Cirrhosis	0.4448	0.4422	0.1304	0.1378	0.2932	0.3117
Diabetes	0.3334	0.2978	0.1446	0.1089	0.4337	0.3656
Cancer	0.1557	0.1436	0.1120	0.0899	0.7195	0.6259
Diarrhea	0.6791	0.6207	0.0979	0.0734	0.1442	0.1183
Digestive	0.5771	0.5724	0.0521	0.0379	0.0903	0.0662
Forces	0.5036	0.4870	0.0359	0.0627	0.0713	0.1287
Maternal	0.7741	0.7666	0.1768	0.1100	0.2284	0.1435
Mental	0.0323	0.0730	0.0080	0.0576	0.2492	0.7887
Musculoskeletal	0.2096	0.2500	0.1557	0.2124	0.7429	0.8497
NTDs + Malaria	0.6733	0.6581	0.1124	0.0934	0.1669	0.1419
Neurological	0.1203	0.0467	0.0143	0.0021	0.1188	0.0456
Nutrition	0.5187	0.5314	0.0668	0.0423	0.1288	0.0796
Other Communicable	0.6118	0.6165	0.1078	0.0832	0.1762	0.1350
Other NCDs	0.3067	0.4245	0.1671	0.1404	0.5450	0.3308
Violence	0.0415	0.1428	0.0096	0.0249	0.2313	0.1741
Transport	0.2832	0.4843	0.2212	0.2812	0.7809	0.5807
Falls + Fire	0.6246	0.6349	0.1233	0.0666	0.1975	0.1049
TB	0.7`05	0.7321	0.1925	0.1750	0.2709	0.2391
HIV	0.2120	0.4271	0.0168	0.0471	0.0791	0.1103

Table 2.2.3. Explained Changes in Mortality Due to Democracy and Other Determinants of Health: Proportions of Variance for Cardiovascular Disease, 1995-2015

Year	GDP per capita	Urbanicity	DAH per capita	Mortality Shock	Democracy	Total Explained by Model (R-squared)	Share of R-squared Attributable to Democracy
1995	0.0525	0.0271	0.0007	0.0019	0.1443	0.2265	0.6373
1996	0.0624	0.0321	0.0097	0.0088	0.1644	0.2773	0.5927
1997	0.0669	0.0386	0.0191	0.0020	0.1726	0.2991	0.5769
1998	0.0688	0.0413	0.0034	0.0092	0.1756	0.2983	0.5888
1999	0.0727	0.0455	0.0030	0.0065	0.1786	0.3064	0.5830
2000	0.0764	0.0507	0.0059	0.0058	0.1884	0.3272	0.5757
2001	0.0746	0.0515	0.0068	0.0073	0.1910	0.3312	0.5766
2002	0.0787	0.0542	0.0090	0.0057	0.2003	0.3479	0.5756
2003	0.0795	0.0552	0.0113	0.0050	0.2106	0.3616	0.5823
2004	0.0800	0.0557	0.0226	0.0025	0.2205	0.3814	0.5782
2005	0.0763	0.0555	0.0426	0.0057	0.2224	0.4025	0.5525
2006	0.0797	0.0588	0.0332	0.0052	0.2311	0.4080	0.5664
2007	0.0820	0.0599	0.0362	0.0093	0.2351	0.4223	0.5567
2008	0.0952	0.0699	0.0137	0.0048	0.2478	0.4315	0.5744
2009	0.1013	0.0715	0.0220	0.0116	0.2474	0.4537	0.5453
2010	0.1103	0.0742	0.0349	0.0173	0.2567	0.4933	0.5204
2011	0.1117	0.0773	0.0335	0.0076	0.2584	0.4885	0.5289
2012	0.1145	0.0791	0.0287	0.0087	0.2525	0.4835	0.5223
2013	0.1221	0.0834	0.0292	0.0045	0.2619	0.5011	0.5226
2014	0.1291	0.0885	0.0175	0.0111	0.2582	0.5045	0.5119
2015	0.1331	0.0883	0.0133	0.0179	0.2523	0.5048	0.4997

Part 2.3: Structural Equation Model
To examine the mediating factors of democracy on health, we estimated a structural equation model (SEM). SEMs allow estimation of the direct effect of variables on outcomes, as performed in typical OLS settings. Additionally, SEMs allow estimation of indirect effects of variables through mediating factors, which then also influence the outcome. For example, we might expect that democracy influences health outcomes directly by increasing media transparency, which can report on quality of health services. However, democracy might also indirectly influence health outcomes through increased government health expenditure, wherein democracies increase spending, and that spending improves health outcomes.

We estimate a long difference specification using Stata's --sem-- command, differencing our variables over 20 years (1995 - 2015) due to the scale of time at which these changes and impacts may occur. Although this reduces the amount of data available to identify the effect of democracy, it captures long-term political, economic, and epidemiological shifts. As is common in difference modeling, and to capture different initial conditions in epidemiology in 1995, we also include a convergence term for the change in mortality rates.[32] A convergence term model makes the first derivative a function of the initial level, so we model changes in mortality rate as a function of the initial level of country mortality in 1995.

For each Level Two cause, we estimate an SEM of the form:

$$\Delta(\text{Government Health Expenditure as Source}_{it}) = \beta_{10} + \beta_{11}\Delta(\text{Democratic Stock}_{it}) + \varepsilon_{it}$$
$$\%\Delta(\text{Urbanicity}_{it}) = \beta_{20} + \beta_{21}\Delta(\text{Democratic Stock}_{it}) + \varepsilon_{it}$$
$$\%\Delta(\text{GDP per capita}_{it}) = \beta_{30} + \beta_{31}\Delta(\text{Democratic Stock}_{it}) + \varepsilon_{it}$$
$$\begin{aligned}\%\Delta(\text{Death Rate}_{it}) = \beta_{40} + \beta_{41}&\Delta(\text{Democratic Stock}_{it}) + \\ &\beta_{42}\Delta(\text{Government Health Expenditure as Source}_{it}) + \\ &\beta_{43}\%\Delta(\text{Urbanicity}_{it}) \\ &\beta_{44}\%\Delta(\text{GDP per capita}_{it}) \\ &\beta_{45}\Delta(\text{Skilled Birth Attendance}_{it}) + \\ &\beta_{46}\Delta(\text{Maternal Education}_{it}) + \\ &\beta_{47}\Delta(\text{Obesity Prevalence}_{it}) + \\ &\beta_{48}\,\text{Death Rate}_{it} + \varepsilon_{it}\end{aligned}$$

Where i is country, t is year, and Δ denotes difference over 20 years. For this analysis, we used data from 165 countries, observing data from 1995 and 2015, and taking the difference. Five countries were dropped from the analysis due to missing data in recent years.

Note that some variables are modeled as absolute changes and others are modeled as percent changes. The most important instance of this is government health expenditure as source (in absolute changes) and GDP per capita (in percent changes). If we modeled both GDP per capita and GHES per capita in absolute changes, then GHES per capita is clearly in the causal pathway (as the amount of GDP per capita has a strong relationship to the absolute amount of health resources sourced from the government). If we modeled both GDP per capita and GHES per capita in percent changes, then GHES per capita is clearly in the causal pathway (as the percent growth in GHES is often correlated with the percent growth in GDP per capita). The percent change in GDP per capita models the economic growth of the country, which slightly informs the dollar change in GHES per capita, but not as directly. For instance, two countries could have the same percent economic growth, but very different resources per person for health.

Figure 2.3.1: Absolute and Percent Changes in GDP and GHES

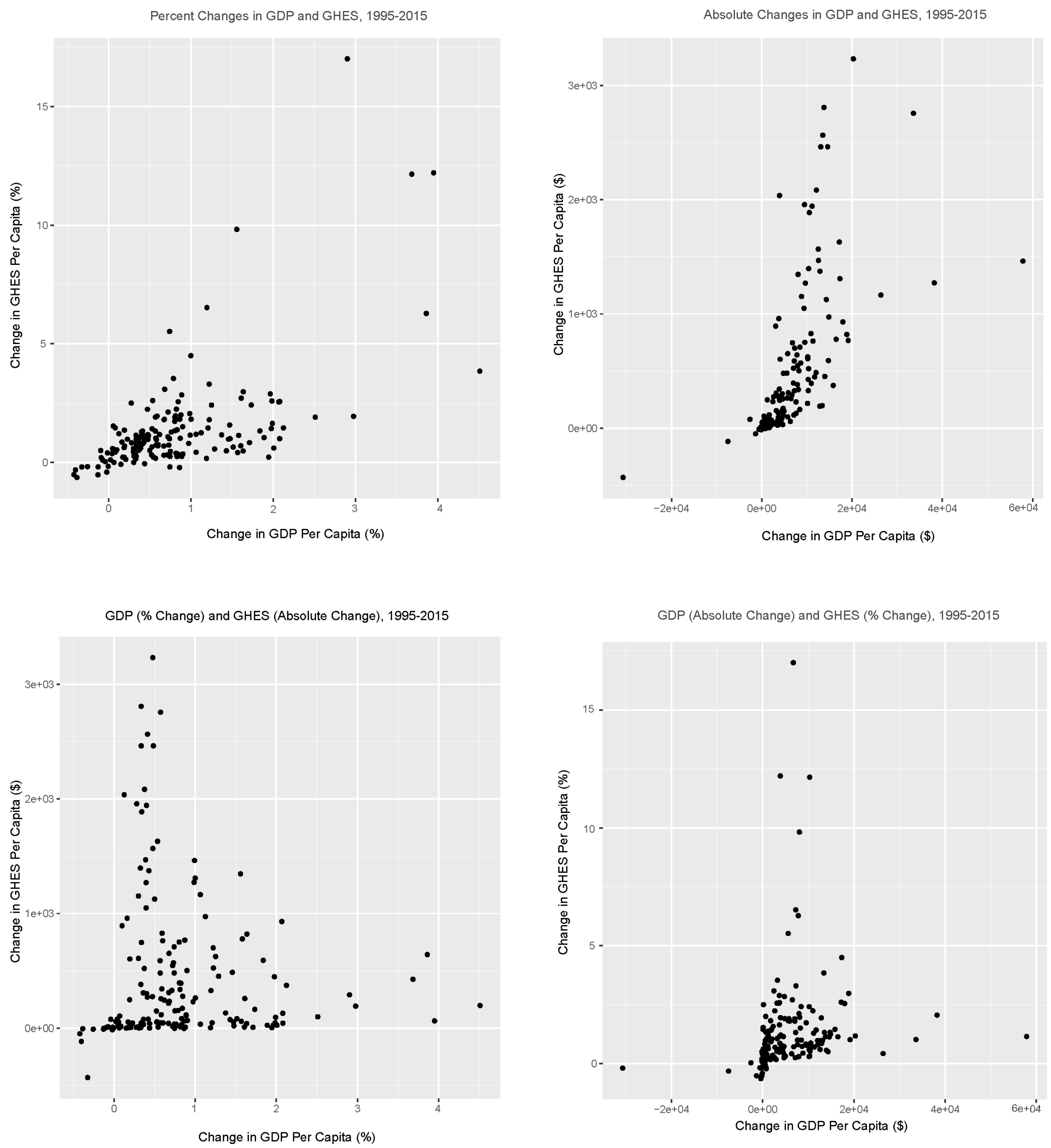

In our model, we aimed to investigate the direct and indirect effects of democracy on health. Thus Figure 4 displays the direct and indirect effects of democracy on health. It is important to note that we display the indirect effect of GDP through democracy on health, where we consider how democracy drives economic development as a percent change which influences health. This model does not display the effect of overall levels of development, because GDP per capita does not enter the model as a level. Figure 2.3.3 considers how fixed geographic attributes (such as past, initial levels of economic development) affect our results.

The following tables displays how to calculate direct and indirect effects:

Table 2.3.1: Direct and Indirect Effects Calculations

Description of Effect	Calculation
Direct effect of changes in democratic stock on changes in age-standardized death rates	β_{41}
Indirect effect of changes in democratic stock on changes in age-standardized death rates	$\beta_{42} * \beta_{11} + \beta_{43} * \beta_{21} + \beta_{44} * \beta_{31}$
Effect of changes in GDP per capita attributable to democratic stock on changes in age-standardized death rates	$\beta_{44} * \beta_{31}$
Effect of changes in urbanization attributable to democratic stock on changes in age-standardized death rates	$\beta_{43} * \beta_{21}$
Effect of changes in government health expenditure attributable to democracy on changes in age-standardized death rates	$\beta_{42} * \beta_{11}$
Total effect of democratic stock on age-standardized death rates	$\beta_{41} + \beta_{42} * \beta_{11} + \beta_{43} * \beta_{21} + \beta_{44} * \beta_{31}$

Figure 2.3.2: Path Diagram of Structural Equation Model

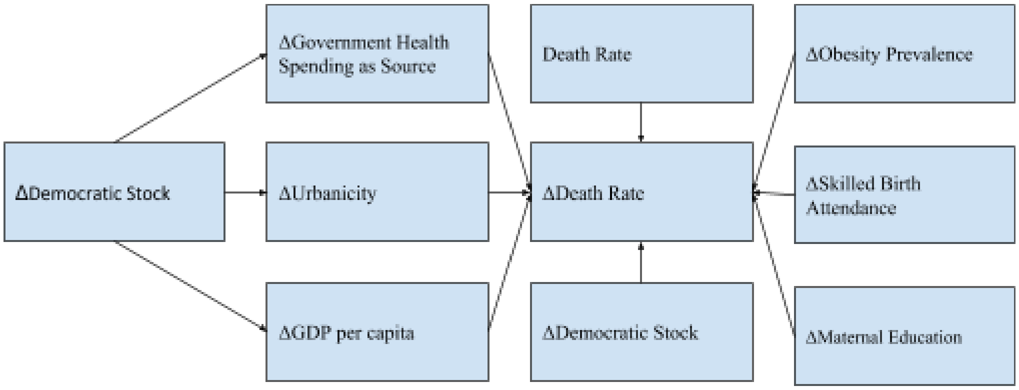

Finally, to address concerns about omitted variable bias due to geography, we also run the model presented in Figure 2.3.2 with fixed effects for GBD super region. Full convergence was not achieved in the maximum likelihood estimation due to a very low (1e-12) tolerance on step size, and so standard errors and p-values should be interpreted with caution. However, the point estimates and effect directions remain very similar controlling for geography, as shown in Figure 2.3.3.

Figure 2.3.3: Structural Equation Model with Geography Fixed Effects

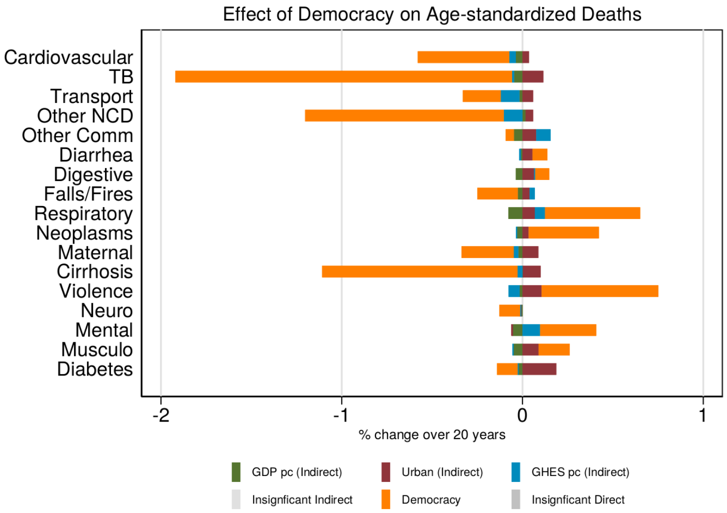

Table 2.3.4: Direct, Indirect, and Total Effects of Democracy
Note: All tables first report coefficient estimates followed by the associated standard errors in brackets.

Panel A: Direct and Indirect Effects of a One-Point Increase in Democracy, 1995-2015

Disease	Dem on Mortality (Indirect)		Dem on Mortality (Direct)		GDP on Mortality (Direct)		GHES on Mortality (Direct)		Urban on Mortality (Direct)	
Cardiovascular	-1.02138	[0.20574]	-0.95344	[0.296149]	-0.00041	[0.000174]	-0.01272	[0.002378]	0.011592	[0.047168]
Cirrhosis	-0.29639	[0.275637]	-0.9213	[0.464464]	0.00026	[0.000278]	-0.01163	[0.003708]	-0.20779	[0.076759]
Diabetes	-0.24226	[0.302235]	0.125634	[0.554344]	-0.00029	[0.000332]	-0.00789	[0.004327]	-0.26338	[0.092466]
Diarrhea	0.476521	[0.242042]	0.388584	[0.413362]	-0.00042	[0.000245]	0.008883	[0.00324]	-0.07563	[0.071739]
Digestive	-0.17821	[0.199347]	0.177081	[0.360502]	-0.00048	[0.000217]	-0.00121	[0.002863]	-0.08512	[0.062934]
Falls + Fire	-0.20832	[0.182383]	-0.28418	[0.306297]	-0.00059	[0.000183]	0.002316	[0.002524]	0.047007	[0.052523]
Forces	-66.6775	[332.627]	-926.437	[747.6024]	-0.26925	[0.446559]	2.165471	[5.684914]	48.57401	[125.2874]
HIV	-19.3119	[14.35116]	17.53766	[31.3693]	0.001747	[0.01887]	-0.2224	[0.240887]	3.995548	[5.233673]
Maternal	0.410921	[0.371604]	0.697381	[0.699874]	-0.00068	[0.000412]	0.009334	[0.005325]	-0.09846	[0.120401]
Mental	0.302603	[0.399291]	-0.14668	[0.862805]	-0.00061	[0.000522]	0.008857	[0.006633]	-0.02408	[0.142581]
Musculoskeletal	-0.28587	[0.348801]	1.141641	[0.734281]	-0.00049	[0.000437]	-0.00526	[0.005813]	-0.18775	[0.118621]
Cancer	-0.44182	[0.126183]	0.106601	[0.235479]	-0.00038	[0.000139]	-0.00432	[0.001773]	-0.01114	[0.038321]
Neurological	-0.07470	[0.067797]	-0.2462	[0.138605]	-8.6E-05	[8.31E-05]	0.000262	[0.001141]	0.032828	[0.022683]
NTDs	1.17680	[0.974397]	0.02245	[1.883469]	0.002564	[0.001124]	-0.01042	[0.014419]	-0.39357	[0.319717]
Nutrition	0.823128	[0.52906]	1.486886	[1.054301]	-0.00083	[0.000627]	0.018437	[0.008001]	-0.03649	[0.173364]
Other communicable	0.397233	[0.282426]	0.139497	[0.497879]	-0.0007	[0.000295]	0.010005	[0.003816]	-0.0676	[0.083032]
Other NCDs	-1.0441	[0.277265]	-0.82852	[0.477628]	-2.2E-05	[0.000281]	-0.0153	[0.003625]	0.041992	[0.078568]
Respiratory	-0.11095	[0.248925]	0.365276	[0.382518]	-0.00113	[0.000227]	0.0058	[0.002921]	-0.04502	[0.064115]
TB	-0.61776	[0.352689]	-1.21159	[0.686812]	-0.00083	[0.000405]	-0.00634	[0.005187]	-0.1207	[0.119861]
Transport	-1.35564	[0.310445]	-0.5913	[0.488937]	-0.00035	[0.000289]	-0.01885	[0.003722]	-0.0047	[0.080738]
Violence	-0.66429	[0.2396]	0.770298	[0.455255]	-0.00049	[0.00027]	-0.00904	[0.003501]	-0.09534	[0.073778]

Panel B: Direct Effects of Democracy on GDP per capita, GHES, and urbanicity

Effect	Effect Size
Democracy on GDP per capita	483.59 [146.00]
Democracy on Urbancity	-1.52 [0.49]
Democracy on GHES	63.37 [11.44]

Panel C: Total Effects of Democracy on Mortality

Disease	Dem on Mortality (Total)	
Cardiovascular	-1.97481	[0.340838]
Cirrhosis	-1.21769	[0.4909079]
Diabetes	-0.11662	[0.5720934]
Diarrhea	0.865104	[0.4503594]
Digestive	-0.00113	[0.3753386]
Falls + Fire	-0.4925	[0.330689]
Forces	-993.114	[730.2446]
HIV	-1.77422	[30.62824]
Maternal	1.108302	[0.7482914]
Mental	0.155927	[0.8428793]
Musculoskeletal	0.855772	[0.7436509]
Cancer	-0.33522	[0.2420971]
Neurological	-0.3209	[0.1334275]
NTDs	1.199245	[1.937382]
Nutrition	2.310014	[1.073083]
Other communicable	0.53673	[0.5348423]
Other NCDs	-1.87263	[0.5100099]
Respiratory	0.254325	[0.4203205]

TB	-1.82935	[0.7158226]
Transport	-1.94695	[0.5328746]
Violence	0.106013	[0.4531403]

Table 2.3.5: Correlations Between Democratic Experience and GDP Per Capita, Government Health Expenditure, and Cardiovascular Mortality by Income Group
Note: these tables display Pearson correlation coefficients and associated p-values in brackets
Panel A: High-income countries

	Democracy	GDP per capita	GHES per capita	Cardio Deaths
Democracy	1			
GDP per capita	0.3722 [0.0084]	1		
GHES per capita	0.0855 [0.5593]	-0.0650 [0.6537]	1	
Cardio Deaths	-0.3392 [0.0171]	-0.1239 [0.3913]	-0.3598 [0.0103]	1

Panel B: Upper-middle Income Countries

	Democracy	GDP per capita	GHES per capita	Cardio Deaths
Democracy	1			
GDP per capita	-0.3738 [0.0148]	1		
GHES per capita	0.0114 [0.9430]	0.2208 [0.1403]	1	
Cardio Deaths	-0.0798 [0.6152]	-0.3364 [0.0223]	-0.2421 [0.1051]	1

Panel C: Lower-middle Income Countries

	Democracy	GDP per capita	GHES per capita	Cardio Deaths
Democracy	1			
GDP per capita	-0.0964 [0.5240]	1		
GHES per capita	0.1379 [0.3607]	0.2208 [0.1403]	1	
Cardio Deaths	0.1745 [0.2461]	-0.3364 [0.0223]	-0.2421 [0.1051]	1

Panel D: Low-income Countries

	Democracy	GDP per capita	GHES per capita	Cardio Deaths
Democracy	1			
GDP per capita	0.1470 [0.4383]	1		
GHES per capita	0.1578 [0.4049]	0.4704 [0.0087]	1	
Cardio Deaths	0.1294 [0.4956]	-0.2984 [0.1092]	-0.2095 [0.2666]	1

Part 2.4: Leave-one-out Analysis
We conduct a "leave-one-out" analysis to examine the impact of each of five components of democracy in reducing cause-specific age-standardized death rates. The "leave-one-out" analysis is termed such because it calculates the Multiplicative Polyarchy Index systematically leaving out one component of democracy, such that we have six democratic stock indices:

1) Democracy
2) Democracy Excluding Suffrage
3) Democracy Excluding Free and Fair Elections
4) Democracy Excluding Free Expression
5) Democracy Excluding Free Association
6) Democracy Excluding Elected Executive

We look at the impact of the stock version of each component by estimating the effect of democratic stock on cause-specific age-standardized mortality rates for each Level Two cause in the following regression framework:

$$\begin{aligned} Death\ rate_{it} = \beta_0 + \beta_1\ (Democratic\ Stock_{it}) + \beta_2\ ln(GDP\ per\ capita_{it}) \\ + \beta_3\ ln(Urbanicity_{it}) + \beta_4\ (Mortality\ Shock_{it}) + \beta_5\ (DAH\ per\ capita_{it}) \\ + \Sigma\ \tau_t 1(year) + \Sigma\ \delta_i 1(iso) + \epsilon_{it} \end{aligned}$$

Where there are i countries and t years. The Democratic Stock variable can be one of six versions listed above. We use Stata's --xtreg-- command to estimate a fixed effects specification with robust standard errors. Our panel data are at the country-year level. We calculate 99% confidence intervals by multiplying the standard error from the regression by 2.58.

To address concerns about omitted variables due to time, we tested both linear year trends and time dummies. All results below use time dummies due to the less stringent assumption on the trend of time.

We include the regressions results of all Level Two causes and HIV and tuberculosis for the leave-one-out analysis below. Column numbers refer to the democratic stock calculation above used as the outcome.

Table 2.4.1: Leave-one-out Analysis Results for All Level Two Causes, HIV, and Tuberculosis and the Sub-components of Democracy

HIV

	**(1)**	**(2)**	**(3)**	**(4)**	**(5)**	**(6)**
All components included	-0.0730***					
	(0.02541)					
Excluding free and fair elections		-0.0432*				
		(0.02271)				
Excluding free expression			-0.0586**			
			(0.02310)			
Excluding free association				-0.0638***		
				(0.02309)		

Excluding suffrage					-0.0670***	
					(0.02483)	
Excluding elected officials						-0.0720***
						(0.02455)
GDP per capita (ln)	0.3205**	0.3426**	0.3404**	0.3305**	0.3335**	0.3295**
	(0.16173)	(0.16638)	(0.16073)	(0.16053)	(0.16121)	(0.16026)
Urbanicity (ln)	-0.1292	-0.0905	-0.1242	-0.1370	-0.1269	-0.1231
	(0.14532)	(0.14935)	(0.14453)	(0.14184)	(0.14436)	(0.14625)
Mortality shock	-0.0004	-0.0012	-0.0003	-0.0003	-0.0005	-0.0004
	(0.00058)	(0.00354)	(0.00342)	(0.00340)	(0.00340)	(0.00338)
DAH per capita	-0.0005	-0.00006	-0.0003	-0.0005	-0.0005	-0.0005
	(0.00096)	(0.00103)	(0.00098)	(0.00097)	(0.00097)	(0.00097)
Constant	-0.7463	-1.091	-0.9456	-0.7621	-0.8784	-0.7629
	(1.59878)	(1.70015)	(1.58434)	(1.59384)	(1.59342)	(1.57878)
Observations	3280	3280	3280	3280	3280	3280
Countries	164	164	164	164	164	164
Country fixed effects	YES	YES	YES	YES	YES	YES
Year fixed effects	YES	YES	YES	YES	YES	YES

*Notes: Cluster-robust standard errors reported in parentheses. * p<0.10 ** p<0.05 *** p<0.01 **** p<0.001*

Tuberculosis

	**(1)**	**(2)**	**(3)**	**(4)**	**(5)**	**(6)**
All components included	-0.0282***					
	(0.00909)					
Excluding free and fair elections		-0.0107				
		(0.00954)				
Excluding free expression			-0.0239***			
			(0.00890)			

Excluding free association				-0.0263***		
				(0.00844)		
Excluding suffrage					-0.0273***	
					(0.00896)	
Excluding elected officials						-0.0286***
						(0.0091)
GDP per capita (ln)	-0.4182****	-0.4038****	-0.4115****	-0.4157****	-0.4144****	-0.4154****
	(0.06612)	(0.06855)	(0.06657)	(0.06613)	(0.06614)	(0.06595)
Urbanicity (ln)	0.1300	0.1444	0.1312	0.1259	0.1301	0.1320
	(0.09145)	(0.10652)	(0.09345)	(0.09109)	(0.00114)	(0.09177)
Mortality shock	-0.0008	-0.0013	-0.0009	-0.0008	-0.0009	-0.0008
	(0.00115)	(0.00119)	(0.00116)	(0.00113)	(0.00115)	(0.00114)
DAH per capita	0.0027***	0.0029***	0.0028***	0.0027***	0.0027***	0.0027***
	(0.00086)	(0.00091)	(0.00087)	(0.00086)	(0.0086)	(0.00085)
Constant	6.0470****	5.7576****	5.9983****	6.0784****	6.0262****	6.0577****
	(0.65529)	(0.71735)	(0.6653)	(0.65713)	(0.65484)	(0.65305)
Observations	3280	3280	3280	3280	3280	3280
Countries	164	164	164	164	164	164
Country fixed effects	YES	YES	YES	YES	YES	YES
Year fixed effects	YES	YES	YES	YES	YES	YES

*Notes: Cluster-robust standard errors reported in parentheses. * p<0.10 ** p<0.05 *** p<0.01 **** p<0.001*

I. Communicable

Diarrhea, Lower Respiratory, and Other Common Infectious Diseases

	**(1)**	**(2)**	**(3)**	**(4)**	**(5)**	**(6)**
All components included	0.0121*					
	(0.00650)					
Excluding free and fair elections		0.0064				

		(0.00672)				
Excluding free expression			0.0108*			
			(0.00587)			
Excluding free association				0.0118**		
				(0.00571)		
Excluding suffrage					0.0119**	
					(0.00635)	
Excluding elected officials						0.0118**
						(0.00632)
GDP per capita (ln)	-0.2999****	-0.3043****	-0.3023****	-0.3004****	-0.3014****	-0.3015****
	(0.03845)	(0.03980)	(0.03851)	(0.0382)	(0.03841)	(0.03849)
Urbanicity (ln)	-0.0306	-0.0370	-0.0309	-0.0285	-0.0306	-0.0317
	(0.12553)	(0.13144)	(0.12573)	(0.12562)	(0.12568)	(0.12588)
Mortality shock	0.0002	0.0004	0.0002	0.0002	0.0002	0.0002
	(0.00076)	(0.00076)	(0.00076)	(0.00074)	(0.00076)	(0.00076)
DAH per capita	-0.0002	-0.0002	-0.0002	-0.0002	-0.0002	-0.0002
	(0.00024)	(0.00025)	(0.00024)	(0.0002)	(0.00024)	(0.00024)
Constant	6.5441****	6.6215****	6.5521****	6.518****	6.5496****	6.547****
	(0.39437)	(0.42536)	(0.3968)	(0.39186)	(0.39383)	(0.039508)
Observations	3280	3280	3280	3280	3280	3280
Countries	164	164	164	164	164	164
Country fixed effects	YES	YES	YES	YES	YES	YES
Year fixed effects	YES	YES	YES	YES	YES	YES

*Notes: Cluster-robust standard errors reported in parentheses. * p<0.10 ** p<0.05 *** p<0.01 **** p<0.001*

Neglected Tropical Diseases and Malaria

	**(1)**	**(2)**	**(3)**	**(4)**	**(5)**	**(6)**
All components included	0.0244**					

	(0.01034)					
Excluding free and fair elections		0.0234**				
		(0.01015)				
Excluding free expression			0.0231**			
			(0.00989)			
Excluding free association				0.0207**		
				(0.00928)		
Excluding suffrage					0.0231**	
					(0.01035)	
Excluding elected officials						0.0202*
						(0.01027)
GDP per capita (ln)	-0.0888	-0.0872	-0.0927	-0.0927	-0.0926	-0.0948
	(0.09129)	(0.09070)	(0.09166)	(0.09128)	(0.09154)	(0.09221)
Urbanicity (ln)	-0.0236	-0.0373	-0.234	-0.0214	-0.0241	-0.0272
	(0.10575)	(0.10786)	(0.10489)	(0.10836)	(0.10690)	(0.11012)
Mortality shock	-0.0002	-0.0008	-0.0002	-0.0002	-0.0001	-0.00007
	(0.00176)	(0.00104)	(0.00176)	(0.00176)	(0.00176)	(0.00176)
DAH per capita	-0.0015	-0.0017	-0.0016	-0.0015	-0.0015	-0.0016
	(0.00100)	(0.00104)	(0.00100)	(0.00100)	(0.00100)	(0.0010)
Constant	0.2826	0.1602	0.2716	0.3039	0.3133	0.3679
	(0.84423)	(0.85387)	(0.84810)	(0.83795)	(0.84460)	(0.85388)
Observations	3280	3280	3280	3280	3280	3280
Countries	164	164	164	164	164	164
Country fixed effects	YES	YES	YES	YES	YES	YES
Year fixed effects	YES	YES	YES	YES	YES	YES

*Notes: Cluster-robust standard errors reported in parentheses. * p<0.10 ** p<0.05 *** p<0.01 **** p<0.001*

Maternal Disorders

	(1)	(2)	(3)	(4)	(5)	(6)
All components included	0.0311***					
	(0.01089)					
Excluding free and fair elections		0.0324***				
		(0.01006)				
Excluding free expression			0.0273***			
			(0.01008)			
Excluding free association				0.0272***		
				(0.00990)		
Excluding suffrage					0.0302***	
					(0.01071)	
Excluding elected officials						0.0271***
						(0.01074)
GDP per capita (ln)	-0.4120****	-0.4074****	-0.4186****	-0.4162****	-0.4162****	-0.4185****
	(0.07698)	(0.07739)	(0.0773)	(0.07726)	(0.07726)	(0.07765)
Urbanicity (ln)	0.2296***	0.2119**	0.2287**	0.2330***	0.2294***	0.2256***
	(0.08111)	(0.08191)	(0.08221)	(0.08375)	(0.08175)	(0.08453)
Mortality shock	0.0016	0.0016	0.0016	0.0015	0.00156	0.0016
	(0.00126)	(0.00122)	(0.00125)	(0.00126)	(0.00126)	(0.00126)
DAH per capita	0.0003	0.0001	0.0002	0.0003	0.0003	0.0002
	(0.00068)	(0.00062)	(0.00066)	(0.00067)	(0.00068)	(0.00067)
Constant	3.5168****	3.2937****	3.5504****	3.523****	3.5409****	3.596****
	(0.72640)	(0.74867)	(0.72737)	(0.72811)	(0.72657)	(0.73013)
Observations	3280	3280	3280	3280	3280	3280
Countries	164	164	164	164	164	164
Country fixed effects	YES	YES	YES	YES	YES	YES
Year fixed effects	YES	YES	YES	YES	YES	YES

*Notes: Cluster-robust standard errors reported in parentheses. * p<0.10 ** p<0.05 *** p<0.01 **** p<0.001*

Neonatal Disorders

	**(1)**	**(2)**	**(3)**	**(4)**	**(5)**	**(6)**
All components included	-0.0162**					
	(0.00678)					
Excluding free and fair elections		-0.0067				
		(0.00613)				
Excluding free expression			-0.0156**			
			(0.00624)			
Excluding free association				-0.0156**		
				(0.00616)		
Excluding suffrage					-0.0175***	
					(0.00661)	
Excluding elected officials						-0.0187***
						(0.00662)
GDP per capita (ln)	-0.2239****	-0.2161****	-0.2213****	-0.2228****	-0.2230****	-0.2239****
	(0.04592)	(0.04699)	(0.04522)	(0.04551)	(-0.04540)	(-0.04503)
Urbanicity (ln)	0.0975*	0.1059*	0.0983*	0.0949*	0.0968*	0.0978*
	(0.05321)	(0.06315)	(0.05239)	(0.05312)	(0.05196)	(0.05117)
Mortality shock	0.0004	0.0001	0.0004	0.0004	0.0004	0.0004
	(0.00068)	(0.00070)	(0.00068)	(0.00068)	(0.00068)	(0.00068)
DAH per capita	0.0010**	0.0011**	0.0010**	0.0010**	0.0010**	0.0010**
	(0.00043)	(0.00046)	(0.00044)	(0.00043)	(0.00043)	(0.00043)
Constant	4.839****	4.6857****	4.8509****	4.8670****	4.8618****	4.8888****
	(0.43069)	(0.45326)	(0.42632)	(0.42923)	(0.42496)	(0.42254)
Observations	3280	3280	3280	3280	3280	3280
Countries	164	164	164	164	164	164

Country fixed effects	YES	YES	YES	YES	YES	YES
Year fixed effects	YES	YES	YES	YES	YES	YES

*Notes: Cluster-robust standard errors reported in parentheses. * p<0.10 ** p<0.05 *** p<0.01 **** p<0.001*

Nutritional deficiencies

	**(1)**	**(2)**	**(3)**	**(4)**	**(5)**	**(6)**
All components included	0.0288**					
	(0.01385)					
Excluding free and fair elections		0.0156				
		(0.01167)				
Excluding free expression			0.0246**			
			(0.01240)			
Excluding free association				0.02849**		
				(0.01242)		
Excluding suffrage					0.0306**	
					(0.01367)	
Excluding elected officials						0.0371***
						(0.01355)
GDP per capita (ln)	-0.2779****	-0.2880***	-0.2845****	-0.2790****	-0.2798****	-0.2747****
	(0.08477)	(-0.08921)	(0.08573)	(0.08484)	(0.08496)	(0.08499)
Urbanicity (ln)	0.1664	0.1512	0.1652	0.1717	0.1675	0.1675
	(0.10472)	(0.09473)	(0.10348)	(0.10491)	(0.10641)	(0.11158)
Mortality shock	0.0015	0.0019	0.0016	0.0014	0.0015	0.0013
	(0.00210)	(0.00221)	(0.00211)	(0.00208)	(0.00209)	(0.00207)
DAH per capita	-0.0004	-0.0005	-0.0004	-0.0004	-0.0004	-0.0003
	(0.00037)	(0.00039)	(0.00037)	(0.00037)	(0.00037)	(0.00038)
Constant	3.1040****	3.2772****	3.1493****	3.0349****	3.0726****	2.9338****
	(0.77765)	(0.86562)	(0.78599)	(0.77875)	(0.77691)	(0.79135)

Observations	3280	3280	3280	3280	3280	3280
Countries	164	164	164	164	164	164
Country fixed effects	YES	YES	YES	YES	YES	YES
Year fixed effects	YES	YES	YES	YES	YES	YES

*Notes: Cluster-robust standard errors reported in parentheses. * p<0.10 ** p<0.05 *** p<0.01 **** p<0.001*

Other Communicable, Maternal, Neonatal, and Nutritional Diseases

	**(1)**	**(2)**	**(3)**	**(4)**	**(5)**	**(6)**
All components included	0.0059					
	(0.00830)					
Excluding free and fair elections		0.0070				
		(0.00710)				
Excluding free expression			0.0043			
			(0.00762)			
Excluding free association				0.0049		
				(0.00738)		
Excluding suffrage					0.0071	
					(0.00821)	
Excluding elected officials						0.0074
						(0.00802)
GDP per capita (ln)	-0.2620****	-0.2603****	-0.2638****	-0.2630****	-0.2617****	-0.2614****
	(0.05548)	(0.05568)	(0.05543)	(0.055407)	(0.05541)	(0.05532)
Urbanicity (ln)	0.1827****	0.1793****	0.1821****	0.1832****	0.1833****	0.1828****
	(0.04095)	(0.04144)	(0.18209)	(0.04138)	(0.04018)	(0.04015)
Mortality shock	-0.00101	-0.00102	-0.00100	-0.0010	-0.0010	-0.0011
	(0.00080)	(0.00080)	(0.00080)	(0.00080)	(0.00080)	(0.00079)
DAH per capita	-0.00002	-0.00007	-0.00004	-0.00003	-0.00002	-0.00002
	(0.00029)	(0.00030)	(0.00029)	(0.00029)	(0.00029)	(0.00030)

Constant	3.2884****	3.2239	3.3125****	3.2951****	3.2643****	3.257****
	(0.52144)	(0.53964)	(0.52208)	(0.5220)	(0.52129)	(0.51994)
Observations	3280	3280	3280	3280	3280	3280
Countries	164	164	164	164	164	164
Country fixed effects	YES	YES	YES	YES	YES	YES
Year fixed effects	YES	YES	YES	YES	YES	YES

*Notes: Cluster-robust standard errors reported in parentheses. * p<0.10 ** p<0.05 *** p<0.01 **** p<0.001*

II. Non-communicable

Neoplasms

	**(1)**	**(2)**	**(3)**	**(4)**	**(5)**	**(6)**
All components included	-0.0061***					
	(0.00212)					
Excluding free and fair elections		-0.00005				
		(0.00215)				
Excluding free expression			-0.0049**			
			(0.00200)			
Excluding free association				-0.0055***		
				(0.00193)		
Excluding suffrage					-0.0057***	
					(0.00210)	
Excluding elected officials						-0.0055***
						(0.00209)
GDP per capita (ln)	-0.0255	-0.0201	-0.0239	-0.0248	-0.0245	-0.0244
	(0.01678)	(0.01710)	(0.01688)	(0.01680)	(0.01680)	(0.01682)
Urbanicity (ln)	0.0457**	0.0486*	0.0461**	0.0449**	0.0458**	0.0464**
	(0.02165)	(0.02636)	(0.02221)	(0.02177)	(0.02196)	(0.02221)
Mortality shock	0.0007**	0.0006**	0.0007**	0.0007***	0.0007**	0.0007**

	(0.00028)	(0.00028)	(0.00028)	(0.00028)	(0.00028)	(0.00023)
DAH per capita	0.0005**	0.0006**	0.0005**	0.0005**	0.0005**	0.0005**
	(0.00023)	(0.00023)	(0.00023)	(0.00023)	(0.00023)	(0.00023)
Constant	5.1832****	5.060*****	5.167****	5.1850****	5.1733****	5.1718****
	(0.15716)	(0.16836)	(0.15844)	(0.15707)	(0.15695)	(0.15681)
Observations	3280	3280	3280	3280	3280	3280
Countries	164	164	164	164	164	164
Country fixed effects	YES	YES	YES	YES	YES	YES
Year fixed effects	YES	YES	YES	YES	YES	YES

*Notes: Cluster-robust standard errors reported in parentheses. * p<0.10 ** p<0.05 *** p<0.01 **** p<0.001*

Cardiovascular Deaths

	**(1)**	**(2)**	**(3)**	**(4)**	**(5)**	**(6)**
All components included	-0.02357****					
	(0.00407)					
Excluding free and fair elections		-0.0076*				
		(0.00386)				
Excluding free expression			-0.0192****			
			(0.00385)			
Excluding free association				-0.0204****		
				0.00374		
Excluding suffrage					-0.0224****	
					(0.00403)	
Excluding elected officials						-0.0233****
						(0.00398)
GDP per capita (ln)	-0.0643**	-0.0509*	-0.0581*	-0.0610*	-0.06073*	-0.0616*
	(0.03227)	(0.03330)	(0.03292)	(0.03277)	(0.03267)	(0.00398)

Urbanicity (ln)	0.0076	0.0196	0.0091	0.0052	0.0080	0.0095
	(0.03534)	(0.04880)	(0.03728)	(0.03732)	(0.03623)	(0.03569)
Mortality shock	0.0005	0.0001	0.0005	0.0006	0.0005	0.0005
	(0.00045)	(0.00049)	(0.00046)	(0.00044)	(0.0005)	(0.00045)
DAH per capita	0.0011***	0.0013***	0.0012***	0.0011***	0.0011***	0.0011***
	(0.00037)	(0.00042)	(0.00038)	0.00038	(0.000374)	(0.00037)
Constant	6.5431****	6.2636****	6.4849****	6.5338****	6.5150****	6.542****
	(0.29296)	(0.30750)	(0.29800)	(0.29621)	(0.29560)	(0.294830)
Observations	3280	3280	3280	3280	3280	3280
Countries	164	164	164	164	164	164
Country fixed effects	YES	YES	YES	YES	YES	YES
Year fixed effects	YES	YES	YES	YES	YES	YES

*Notes: Cluster-robust standard errors reported in parentheses. * p<0.10 ** p<0.05 *** p<0.01 **** p<0.001*

Chronic Respiratory Deaths

	**(1)**	**(2)**	**(3)**	**(4)**	**(5)**	**(6)**
All components included	0.0095					
	(0.00583)					
Excluding free and fair elections		0.0146**				
		(0.00603)				
Excluding free expression			0.0098*			
			(0.00541)			
Excluding free association				0.0102*		
				(0.00541)		
Excluding suffrage					0.0114*	
					(0.0058)	
Excluding elected officials						0.0111*
						(0.00594)

GDP per capita (ln)	-0.2050****	-0.1990****	-0.2059****	-0.2047****	-0.2047****	-0.2049****
	(0.04454)	(0.04428)	(0.04484)	(0.04460)	(0.04473)	(0.04467)
Urbanicity (ln)	0.1270***	0.1212**	0.1275***	0.1293***	0.1280***	0.1269***
	(0.04418)	(0.04763)	(0.04514)	(0.04515)	(0.04568)	(0.04533)
Mortality shock	0.0014	0.0013	0.0013	0.0013	0.0013	0.0013
	(0.00090)	(0.00090)	(0.00090)	(0.00089)	(0.00033)	(0.00089)
DAH per capita	0.0006*	0.0006*	0.0006*	0.0006*	0.0006*	0.0006*
	(0.00032)	(0.00031)	(0.00032)	(0.00033)	(0.00033)	(0.00033)
Constant	5.419****	5.2263****	5.3964****	5.3783****	5.3832****	5.3866****
	(0.40286)	(0.4185)	(0.40551)	(0.40210)	(0.40420)	(0.40170)
Observations	3280	3280	3280	3280	3280	3280
Countries	164	164	164	164	164	164
Country fixed effects	YES	YES	YES	YES	YES	YES
Year fixed effects	YES	YES	YES	YES	YES	YES

*Notes: Cluster-robust standard errors reported in parentheses. * p<0.10 ** p<0.05 *** p<0.01 **** p<0.001*

Cirrhosis and Other Chronic Liver Diseases

	**(1)**	**(2)**	**(3)**	**(4)**	**(5)**	**(6)**
All components included	-0.0066					
	(0.00651)					
Excluding free and fair elections		-0.0019				
		(0.00557)				
Excluding free expression			-0.0057			
			(0.00582)			
Excluding free association				-0.0055		
				(0.00597)		
Excluding suffrage					-0.0058	
					(0.00647)	

Excluding elected officials						-0.0081
						(0.00649)
GDP per capita (ln)	0.0732	0.0772	0.0747	0.0744	0.0746	0.0728
	(0.05222)	(0.05208)	(0.05230)	(0.05227)	(0.05220)	(0.05235)
Urbanicity (ln)	-0.0194	-0.0161	-0.0192	-0.0199	-0.0190	-0.0195
	(0.07080)	(0.06616)	(0.07048)	(0.06989)	(0.07001)	(0.07232)
Mortality shock	-0.0012	-0.0013	-0.0012	-0.0012	-0.0012	-0.0011
	(0.00090)	(0 .00095)	(0.00091)	(0.00090)	(0.00090)	(0.00090)
DAH per capita	0.00001	0.00005	0.00003	0.00002	0.00002	0.00001
	(0.00024)	(0.00025)	(0.00025)	(0.00025)	(0.00024)	(0.00025)
Constant	2.3755****	2.2922****	2.3669****	2.3675****	2.3580****	2.4074****
	(0.48442)	(0.48507)	(0.48285)	(0.48477)	(0.48258)	(0.48531)
Observations	3280	3280	3280	3280	3280	3280
Countries	164	164	164	164	164	164
Country fixed effects	YES	YES	YES	YES	YES	YES
Year fixed effects	YES	YES	YES	YES	YES	YES

*Notes: Cluster-robust standard errors reported in parentheses. * p<0.10 ** p<0.05 *** p<0.01 **** p<0.001*

Digestive Diseases

	**(1)**	**(2)**	**(3)**	**(4)**	**(5)**	**(6)**
All components included	0.0069*					
	(0.00388)					
Excluding free and fair elections		0.0086**				
		(0.00371)				
Excluding free expression			0.0076**			
			(0.00367)			
Excluding free association				0.0074**		
				(0.00363)		

Excluding suffrage					0.0078**	
					(0.00388)	
Excluding elected officials						0.0068*
						(0.00387)
GDP per capita (ln)	-0.0988****	-0.0961****	-0.0989****	-0.0984****	-0.0986****	-0.0994****
	(0.02798)	(0.02794)	(0.02772)	(0.02778)	(0.02780)	(0.02796)
Urbanicity (ln)	0.0804**	0.0765**	0.0811**	0.0822**	0.0811**	0.0800**
	(0.03522)	(0.03464)	(0.03389)	(0.03446)	(0.03423)	(0.03532)
Mortality shock	0.00008	0.00005	0.00003	0.00003	0.00005	0.00007
	(0.00061)	(0.00062)	(0.00062)	(0.00061)	(0.00062)	(0.00061)
DAH per capita	0.0004	0.0003	0.0004	0.0004	0.0004	0.0004
	(0.00023)	(0.00023)	(0.00023)	(0.00023)	(0.00023)	(0.00023)
Constant	3.7819****	3.6894****	3.7519****	3.748****	3.7594****	3.778****
	(0.26661)	(0.28238)	(0.26695)	(0.26765)	(0.26573)	(0.26729)
Observations	3280	3280	3280	3280	3280	3280
Countries	164	164	164	164	164	164
Country fixed effects	YES	YES	YES	YES	YES	YES
Year fixed effects	YES	YES	YES	YES	YES	YES

*Notes: Cluster-robust standard errors reported in parentheses. * p<0.10 ** p<0.05 *** p<0.01 **** p<0.001*

Neurological Disorders

	**(1)**	**(2)**	**(3)**	**(4)**	**(5)**	**(6)**
All components included	-0.0028***					
	(0.00098)					
Excluding free and fair elections		-0.0017*				
		(0.00093)				
Excluding free expression			-0.0027***			
			(0.00097)			

Excluding free association				-0.0024**		
				(0.00096)		
Excluding suffrage					-0.0026**	
					(0.00099)	
Excluding elected officials						-0.0028***
						(0.00097)
GDP per capita (ln)	0.0172***	0.0180***	0.0177***	0.0177***	0.0178***	0.0176***
	(0.00583)	(0.00601)	(0.00586)	(0.00589)	(0.00584)	(0.00581)
Urbanicity (ln)	0.0117	0.0132	0.0117	0.0114	0.0118	0.0119
	(0.01294)	(0.00016)	(0.01287)	(0.01328)	(0.01312)	(0.01297)
Mortality shock	0.0008	0.00005	0.00009	0.00009	0.00008	0.00008
	(0.00016)	(0.00009)	(0.00016)	(0.00016)	(0.00016)	(0.00016)
DAH per capita	0.0002	0.0002**	0.0002**	0.0002*	0.0002*	0.0002*
	(0.00008)	(0.00009)	(0.00008)	(0.00008)	(0.00008)	(0.00008)
Constant	3.6865****	3.6748****	3.6872****	3.6848****	3.6803****	3.6848****
	(0.05070)	(0.05464)	(0.05158)	(0.05212)	(0.05089)	(0.05068)
Observations	3280	3280	3280	3280	3280	3280
Countries	164	164	164	164	164	164
Country fixed effects	YES	YES	YES	YES	YES	YES
Year fixed effects	YES	YES	YES	YES	YES	YES

*Notes: Cluster-robust standard errors reported in parentheses. * p<0.10 ** p<0.05 *** p<0.01 **** p<0.001*

Mental and Substance Use Disorders

	**(1)**	**(2)**	**(3)**	**(4)**	**(5)**	**(6)**
All components included	0.0022					
	(0.00827)					
Excluding free and fair elections		0.0048				
		(0.00804)				

Excluding free expression			0.0005			
			(0.0077)			
Excluding free association				0.0007		
				(0.00751)		
Excluding suffrage					0.0027	
					(0.00806)	
Excluding elected officials						0.00049
						(0.00817)
GDP per capita (ln)	-0.0564	-0.0536	-0.0580	-0.0577	-0.0562	-0.0580
	(0.04940)	(0.04944)	(0.04899)	(0.04910)	(0.04907)	(0.04943)
Urbanicity (ln)	-0.0081	-0.0096	-0.0089	-0.0087	-0.0079	-0.00900
	(0.04020)	(0.03969)	(0.04074)	(0.04067)	(0.04008)	(0.04100)
Mortality shock	-0.0002	-0.0002	-0.0001	-0.0001	-0.0002	-0.00014
	(0.00067)	(0.00038)	(0.00067)	(0.00067)	(0.00067)	(0.00068)
DAH per capita	0.0004	0.0003	0.0004	0.0004	0.0004	0.0004
	(0.00039)	(0.00038)	(0.00039)	(0.00039)	(0.00039)	(0.00039)
Constant	1.6006****	1.5197***	1.635****	1.6286****	1.5907****	1.6357****
	(0.4917()	(0.51530)	(0.49036)	(0.49159)	(0.48946)	(0.49622)
Observations	3280	3280	3280	3280	3280	3280
Countries	164	164	164	164	164	164
Country fixed effects	YES	YES	YES	YES	YES	YES
Year fixed effects	YES	YES	YES	YES	YES	YES

*Notes: Cluster-robust standard errors reported in parentheses. * p<0.10 ** p<0.05 *** p<0.01 **** p<0.001*

Diabetes, Urogenital, Blood, and Endocrine Diseases Deaths

	**(1)**	**(2)**	**(3)**	**(4)**	**(5)**	**(6)**
All components included	-0.003					
	(0.00545)					

Excluding free and fair elections		0.0012				
		(0.00552)				
Excluding free expression			-0.0024			
			(0.03582)			
Excluding free association				-0.0041		
				(0.00483)		
Excluding suffrage					-0.0032	
					(0.00545)	
Excluding elected officials						-0.0024
						(0.00536)
GDP per capita (ln)	-0.0470	-0.0434	-0.0464	-0.0481	-0.0471	-0.0465
	(0.03579)	(0.03683)	(0.03582)	(0.03547)	(0.03572)	(0.03592)
Urbanicity (ln)	0.0176	0.0187	0.0176	0.0161	0.0173	0.0179
	(0.00073)	(0.04154)	(0.03857)	(0.03744)	(0.03572)	(0.03898)
Mortality shock	-0.0008	-0.0009	-0.0008	-0.0008	-0.0008	-0.0008
	(0.00032)	(0.00072)	(0.00073)	(0.00071)	(0.00073)	(0.00072)
DAH per capita	0.0005	0.0005	0.0005	0.0005	0.0005	0.0005
	(0.00032)	(0.00033)	(0.00032)	(0.00032)	(0.00032)	(0.00032)
Constant	4.4998****	4.4122****	4.4964****	4.5390****	4.5099****	4.4941****
	(0.34012)	(0.00257)	(0.34240)	(0.33482)	(0.33912)	(0.34231)
Observations	3280	3280	3280	3280	3280	3280
Countries	164	164	164	164	164	164
Country fixed effects	YES	YES	YES	YES	YES	YES
Year fixed effects	YES	YES	YES	YES	YES	YES

*Notes: Cluster-robust standard errors reported in parentheses. * p<0.10 ** p<0.05 *** p<0.01 **** p<0.001*

Musculoskeletal Disorders

	**(1)**	**(2)**	**(3)**	**(4)**	**(5)**	**(6)**

All components included	-0.0056					
	(0.00682)					
Excluding free and fair elections		0.0043				
		(0.00648)				
Excluding free expression			0.0004			
			(0.00658)			
Excluding free association				-0.0013		
				(0.00648)		
Excluding suffrage					-0.0013	
					(0.00708)	
Excluding elected officials						0.0011
						(0.00714)
GDP per capita (ln)	0.0141	0.0234	0.0194	0.0180	0.0181	0.0199
	(0.04676)	(0.04678)	(0.04720)	(0.04708)	(0.04716)	(0.04769)
Urbanicity (ln)	-0.0279	-0.0256	-0.0250	-0.0261	-0.0259	-0.0248
	(0.06376)	(0.05495)	(0.0577)	(0.05951)	(0.00085)	(0.05767)
Mortality shock	-0.0007	-0.0010	-0.0009	-0.0008	-0.0009	-0.0009
	(0.00084)	(0.00088)	(0.00085)	(0.00084)	(0.00085)	(0.00085)
DAH per capita	0.0002	0.0002	0.0002	0.0002	0.0002	0.0002
	(0.00030)	(0.00031)	(0.00031)	(0.00031)	(0.00031)	(0.00031)
Constant	0.0077	-0.2203	-0.1149	-0.0761	-0.0795	-0.1280
	(0.41830)	(0.4225)	(0.42358)	(0.42283)	(0.42401)	(0.43635)
Observations	3280	3280	3280	3280	3280	3280
Countries	164	164	164	164	164	164
Country fixed effects	YES	YES	YES	YES	YES	YES
Year fixed effects	YES	YES	YES	YES	YES	YES

*Notes: Cluster-robust standard errors reported in parentheses. * p<0.10 ** p<0.05 *** p<0.01 **** p<0.001*

Other Non-communicable Diseases

	**(1)**	**(2)**	**(3)**	**(4)**	**(5)**	**(6)**
All components included	-0.0103****					
	(0.00274)					
Excluding free and fair elections		-0.0018				
		(0.00265)				
Excluding free expression			-0.0082***			
			(0.00262)			
Excluding free association				-0.0092****		
				(0.002495)		
Excluding suffrage					-0.0099****	
					(0.00271)	
Excluding elected officials						-0.0104****
						(0.00269)
GDP per capita (ln)	-0.0736****	-0.0662****	-0.0708****	-0.0724****	-0.0721****	-0.0726****
	(0.01916)	(0.01949)	(0.01934)	(0.01924)	(0.01925)	(0.01922)
Urbanicity (ln)	0.0390*	0.0441*	0.0397*	0.0377*	0.0391*	0.0397*
	(0.02013)	(0.02565)	(0.02080)	(0.02034)	(0.02039)	(0.02028)
Mortality shock	0.0003	0.0001	0.0003	0.0003	0.0003	0.0003
	(0.00030)	(0.00031)	(0.00030)	(0.00029)	(0.00030)	(0.00030)
DAH per capita	0.0007***	0.0008***	0.0007***	0.0007**	0.0007***	0.0007***
	(0.00027)	(0.00029)	(0.00027)	(0.00027)	(0.00027)	(0.00027)
Constant	7.288****	7.1254****	7.2581****	7.2906****	7.2767****	7.2907****
	(0.17927)	(0.18598)	(0.18071)	(0.17885)	(0.17931)	(0.17869)
Observations	3280	3280	3280	3280	3280	3280
Countries	164	164	164	164	164	164
Country fixed effects	YES	YES	YES	YES	YES	YES

Year fixed effects	YES	YES	YES	YES	YES	YES

*Notes: Cluster-robust standard errors reported in parentheses. * p<0.10 ** p<0.05 *** p<0.01 **** p<0.001*

II. Injuries

Transport Injuries

	**(1)**	**(2)**	**(3)**	**(4)**	**(5)**	**(6)**
All components included	-0.0276****					
	(0.00714)					
Excluding free and fair elections		-0.0110*				
		(0.00611)				
Excluding free expression			-0.0223****			
			(0.00651)			
Excluding free association				-0.0257****		
				(0.00630)		
Excluding suffrage					-0.0276****	
					(0.00694)	
Excluding elected officials						-0.0282****
						(0.00685)
GDP per capita (ln)	-0.0214	-0.0077	-0.0139	-0.0189	-0.0182	-0.0187
	(0.04528)	(0.04667)	(0.04594)	(0.04586)	(0.04575)	(0.04585)
Urbanicity (ln)	0.0845**	0.0987*	0.0864**	0.0806**	0.0843**	0.0864**
	(0.03655)	(0.05138)	(0.03919)	(0.03736)	(0.03676)	(0.03695)
Mortality shock	0.0008	0.0004	0.0007	0.0009	0.0008	0.0008
	(0.00066)	(0.0007)	(0.00066)	(0.00065)	(0.00066)	(0.00066)
DAH per capita	0.0015****	0.0016****	0.0015****	0.0015****	0.0015****	0.0015****
	(0.00040)	(0.00046)	(0.00042)	(0.00041)	(0.00041)	(0.00040)
Constant	3.3474****	3.0755****	3.2751****	3.3764****	3.342****	3.3614****
	(0.42290)	(0.45194)	(0.42938)	(0.42666)	(0.42608)	(0.42493)

Observations	3280	3280	3280	3280	3280	3280
Countries	164	164	164	164	164	164
Country fixed effects	YES	YES	YES	YES	YES	YES
Year fixed effects	YES	YES	YES	YES	YES	YES

*Notes: Cluster-robust standard errors reported in parentheses. * p<0.10 ** p<0.05 *** p<0.01 **** p<0.001*

Unintentional Injuries

	(1)	(2)	(3)	(4)	(5)	(6)
All components included	-0.0004					
	(0.00400)					
Excluding free and fair elections		0.0019				
		(0.00369)				
Excluding free expression			-0.00040			
			(0.00371)			
Excluding free association				-0.0006		
				(0.00269)		
Excluding suffrage					-0.0001	
					(0.00399)	
Excluding elected officials						-0.0016
						(0.00386)
GDP per capita (ln)	-0.1446****	-0.1424****	-0.1446****	-0.1448****	-0.1443****	-0.1455****
	(0.02728)	(0.02789)	(0.02719)	(0.02720)	(0.02723)	(0.02723)
Urbanicity (ln)	0.0326	0.03267	0.0326	0.0324	0.0328	0.0322
	(0.02525)	(0.02721)	(0.02524)	(0.02507)	(0.02546)	(0.02724)
Mortality shock	0.0003	0.0002	0.0003	0.0003	0.0003	0.0003
	(0.00059)	(0.00058)	(0.00059)	(0.00059)	(0.00059)	(0.00059)
DAH per capita	0.0006**	0.0006**	0.0006**	0.0006**	0.0006**	0.0006**
	(0.0003)	(0.00028)	(0.00028)	(0.00028)	(0.00028)	(0.00028)

Constant	4.4876****	4.4286****	4.4876****	4.4931****	4.4805****	4.5119***
	(0.25524)	(0.27393)	(0.25497)	(0.25545)	(0.2549)	(0.25570)
Observations	3280	3280	3280	3280	3280	3280
Countries	164	164	164	164	164	164
Country fixed effects	YES	YES	YES	YES	YES	YES
Year fixed effects	YES	YES	YES	YES	YES	YES

*Notes: Cluster-robust standard errors reported in parentheses. * p<0.10 ** p<0.05 *** p<0.01 **** p<0.001*

Self-harm and Interpersonal Violence

	**(1)**	**(2)**	**(3)**	**(4)**	**(5)**	**(6)**
All components included	-0.0001					
	(0.00616)					
Excluding free and fair elections		0.0034				
		(0.00526)				
Excluding free expression			0.0004			
			(0.00562)			
Excluding free association				-0.0009		
				(0.00558)		
Excluding suffrage					0.0003	
					(0.00606)	
Excluding elected officials						-0.0013
						(0.00601)
GDP per capita (ln)	-0.1612****	-0.1567****	-0.1598****	-0.1609****	-0.1599****	-0.1612****
	(0.03295)	(0.03287)	(0.03264)	(0.03269)	(0.03279)	(0.03276)
Urbanicity (ln)	0.0320	0.0318	0.0323	0.0314	0.0322	0.0315
	(0.03251)	(0.03400)	(0.03265)	(0.03223)	(0.03263)	(0.03234)
Mortality shock	0.0002	0.0001	0.0002	0.0002	0.0002	0.0002
	(0.00072)	(0.00072)	(0.00072)	(0.00072)	(0.00072)	(0.00072)

DAH per capita	0.0009***	0.0009***	0.0009***	0.0009***	0.0009***	0.0009***
	(0.00028)	(0.00029)	(0.00028)	(0.00028)	(0.00028)	(0.00028)
Constant	4.1415****	4.0495****	4.1307****	4.1611****	4.1338****	4.167****
	(0.32534)	(0.33282)	(0.32230)	(0.32338)	(0.32404)	(0.32353)
Observations	3280	3280	3280	3280	3280	3280
Countries	164	164	164	164	164	164
Country fixed effects	YES	YES	YES	YES	YES	YES
Year fixed effects	YES	YES	YES	YES	YES	YES

*Notes: Cluster-robust standard errors reported in parentheses. * p<0.10 ** p<0.05 *** p<0.01 **** p<0.001*

Forces of Nature, Conflict and Terrorism, Executions and Police Conflict

	**(1)**	**(2)**	**(3)**	**(4)**	**(5)**	**(6)**
All components included	0.0230					
	(0.03398)					
Excluding free and fair elections		0.0138				
		(0.03379)				
Excluding free expression			0.0130			
			(0.03308)			
Excluding free association				0.0124		
				(0.03169)		
Excluding suffrage					0.0247	
					(0.03345)	
Excluding elected officials						0.0202
						(0.03426)
GDP per capita (ln)	-1.1296***	-1.1364***	-1.1401***	-1.1394***	-1.1309***	-1.1344***
	(0.03398)	(0.36309)	(0.35999)	(0.36074)	(0.36128)	(0.36145)
Urbanicity (ln)	-0.2797	-0.2919	-0.2841	-0.2824	-0.2787	-0.2826
	(0.39816)	(0.40424)	(0.40406)	(0.40642)	(0.39700)	(0.39976)

Mortality shock	-0.2262****	-0.2260****	-0.2260****	-0.2260****	-0.2263****	-0.2262****
	(0.01077)	(0.01073)	(0.01077)	(0.01078)	(0.01078)	(-0.01077)
DAH per capita	-0.0058**	-0.0059**	-0.0059**	-0.0058**	-0.0058**	-0.0058**
	(0.00279)	(0.00279)	(0.00278)	(0.00279)	(0.00278)	(0.00280)
Constant	6.7331*	6.8373*	6.9170**	6.9165**	6.7025*	6.7890*
	(3.4603)	(3.53831)	(3.45775)	(3.46121)	(3.44095)	(3.45377)
Observations	3280	3280	3280	3280	3280	3280
Countries	164	164	164	164	164	164
Country fixed effects	YES	YES	YES	YES	YES	YES
Year fixed effects	YES	YES	YES	YES	YES	YES

*Notes: Cluster-robust standard errors reported in parentheses. * p<0.10 ** p<0.05 *** p<0.01 **** p<0.001*

We also take a closer look at the sub-components that comprise free and fair elections. Those sub-components are (V-Dem label in parenthesis): vote buying (v2elvotbuy), electoral fraud (v2elrgstry & v2elirreg), intimidation of the opposition (v2elintim), and electoral board autonomy and capacity (v2elembaut & v2elembcap). The age-standardized cardiovascular death rate is regressed on the stock version of each of those sub-components, using the previous equation. Those regressions suggest vote buying is the sub-component that is the most negatively associated with cardiovascular mortality, followed by voting irregularities (includes ballot stuffing, false collation of votes, misreporting of votes) and manipulation of the voting registry.

Table 2.4.2: Cardiovascular Deaths and the Sub-components of Free and Fair Elections

	**(1)**	**(2)**	**(3)**	**(4)**	**(5)**	**(6)**
Absence of vote buying	-0.0273****					
	(0.00426)					
Absence of voting irregularities		-0.0241****				
		(0.00424)				
Absence of registration fraud			-0.0225****			
			(0.00472)			
Absence of government intimidation of opposition				-0.0179****		
				(0.00433)		
Electoral board capacity					-0.0156***	

					(0.00511)	
Electoral board autonomy						-0.00373
						(0.00439)
GDP per capita (ln)	-0.0353	-0.0566*	-0.0554	-0.0517	-0.0231	-0.0445
	(0.0363)	(0.0334)	(0.0353)	(0.0333)	(0.0392)	(0.0338)
Urbanicity (ln)	-0.0129	-0.0104	0.0220	0.0188	0.0178	0.0194
	(0.0462)	(0.0416)	(0.0416)	(0.0448)	(0.0421)	(0.0338)
Mortality shock	0.000382	0.000594	0.000450	0.000388	-0.00000266	0.0000497
	(0.000465)	(0.000415)	(0.000426)	(0.000477)	(0.000492)	(0.000477)
DAH per capita	0.00108***	0.00112***	0.00125***	0.00117***	0.00117***	0.00126***
	(0.000361)	(0.000372)	(0.000404)	(0.000376)	(0.000380)	(0.000418)
Constant	6.589****	6.716****	6.679****	6.534****	6.304****	6.175****
	(0.327)	(0.308)	(0.326)	(0.309)	(0.337)	(0.319)
Observations	3280	3280	3280	3280	3280	3280
Countries	164	164	164	164	164	164

*Notes: Cluster-robust standard errors reported in parentheses. * p<0.10 ** p<0.05 *** p<0.01 **** p<0.001*

In addition, we introduce an interaction term in order to assess whether vote buying conditions the reductive effect of government health expenditure on the age-standardized cardiovascular death rate, as follows:

$$
\begin{aligned}
\textit{Cardiovascular death rate}_{it} = {} & \beta_0 + \beta_1 (\textit{Vote Buying Stock}_{it}) \\
& + \beta_2 \ln(\textit{Government Health Expenditure as Source}_{it}) \\
& + \beta_3 \textit{Vote Buying Stock}_{it} \times \ln(\textit{Government Health Expenditure as Source}_{it}) \\
& + \beta_4 \ln(\textit{GDP per capita})_{it} \\
& + \beta_5 \ln(\textit{Urbanicity}_{it}) + \beta_6 (\textit{Mortality Shock}_{it}) + \beta_7(\textit{DAH per capita}_{it}) \\
& \quad + \Sigma \tau_t 1(\textit{year}) + \Sigma \delta_i 1(\textit{iso}) + \epsilon_{it}
\end{aligned}
$$

The results of that model are presented in the following figure, Figure 2.4.1. The x-axis displays the democratic stock that accumulates as vote buying does not occur. The y-axis displays the marginal effect of government health expenditure (as source) on cardiovascular mortality as vote buying decreases. Reductions in vote buying magnify the reductive effect of government health spending on cardiovascular mortality. One hypothesis might be that vote buying leads to less health spending on low-income voters.

Figure 2.4.1: Vote Buying, Government Health Expenditure (GHE), and Cardiovascular Deaths (All Ages)

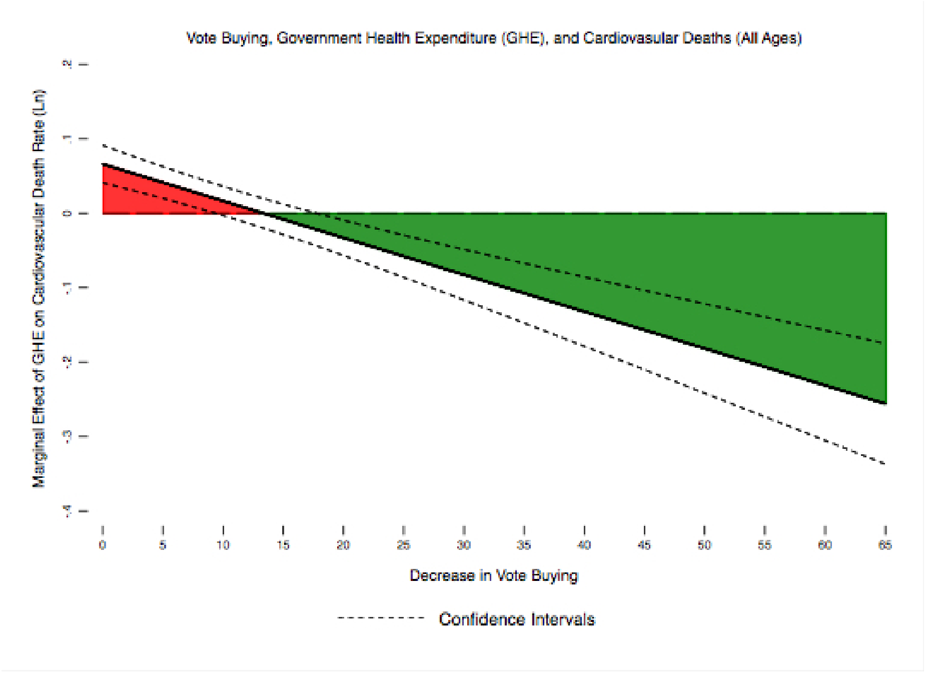

3.0 TABLES

Table 3.1: Classification of Countries by GBD SDI Groupings

High SDI	High-middle SDI	Middle SDI	Low-middle SDI	Low SDI
Andorra	Antigua and Barbuda	Albania	Bangladesh	Afghanistan
Australia	Argentina	Algeria	Belize	Angola
Austria	Armenia	American Samoa	Bhutan	Benin
Belgium	Azerbaijan	Bahrain	Bolivia	Burkina Faso
Canada	Barbados	Bali	Cambodia	Burundi
Croatia	Belarus	Bosnia and Herzegovina	Cameroon	Central African Republic
Cyprus	Bermuda	Botswana	Cape Verde	Chad
Czech Republic	Bulgaria	Colombia	Gabon	Comoros
Denmark	Chile	Costa Rica	Ghana	Cote d'Ivoire
Estonia	Cuba	Dominica	Guatemala	Democratic Republic of the Congo
Finland	Georgia	Dominican Republic	Honduras	Djibouti
France	Guam	Ecuador	Iraq	Eritrea
Germany	Hong Kong Special Administrative Region of China	Egypt	Kyrgyzstan	Ethiopia
Greece	Hungary	El Salvador	Laos	Guinea
Iceland	Iran	Equatorial Guinea	Lesotho	Guinea-Bissau
Ireland	Israel	Fiji	Marshall Islands	Haiti
Italy	Kazakhstan	Grenada	Mauritania	Liberia
Latvia	Kuwait	Guyana	Morocco	Madagascar
Luxembourg	Lebanon	Jamaica	Myanmar	Malawi
Malta	Libya	Jordan	Namibia	Mali
Netherlands	Macao Special Administrative Region of China	Maldives	Nepal	Mozambique
New Zealand	Macedonia	Moldova	Nicaragua	Niger
Northern Ireland	Malaysia	Mongolia	Nigeria	Palestine
Norway	Mauritius	Mexico	North Korea	Papua New Guinea
Poland	Montenegro	Paraguay	Pakistan	Rwanda

Singapore	Panama	Peru	Papua	Sao Tome and Principe
Slovakia	Portugal	Philippines	Samoa	Senegal
Slovenia	Qatar	Saint Lucia	Sudan	Sierra Leone
Sweden except Stockholm	Romania	Saint Vincent and the Grenadines	Swaziland	Solomon Islands
Switzerland	Russia	Seychelles	Syria	Somalia
Taiwan (Province of China)	Serbia	Sri Lanka	Tajikistan	South Sudan
	Spain	Suriname	Tibet	Tanzania
	The Bahamas	Thailand	Timor-Leste	The Gambia
	Trinidad and Tobago	Tunisia	Vanuatu	Togo
	Turkey	Uruguay	Zambia	Uganda
	Turkmenistan	Uzbekistan	Zimbabwe	Yemen
	Ukraine	Venezuela		
	United Arab Emirates	Vietnam		

Table 3.2: Classification of Countries by World Bank Income Groups

**High Income**	**Upper-middle income**	**Lower-middle Income**	**Low Income**
Andorra	Albania	Angola	Afghanistan
Antigua and Barbuda	Algeria	Bangladesh	Benin
Argentina	American Samoa	Bhutan	Burkina Faso
Aruba	Armenia	Bolivia	Burundi
Australia	Azerbaijan	Cabo Verde	Central African Republic
Austria	Belarus	Cambodia	Chad
Bahamas, The	Belize	Cameroon	Comoros
Bahrain	Bosnia and Herzegovina	Congo, Rep.	Congo, Dem. Rep.
Barbados	Botswana	Côte d'Ivoire	Eritrea
Belgium	Brazil	Djibouti	Ethiopia
Bermuda	Bulgaria	Egypt, Arab Rep.	Gambia, The
British Virgin Islands	China	El Salvador	Guinea
Brunei Darussalam	Colombia	Georgia	Guinea-Bissau
Canada	Costa Rica	Ghana	Haiti

Cayman Islands	Cuba	Honduras	Korea, Dem. People's Rep.
Channel Islands	Dominica	India	Liberia
Chile	Dominican Republic	Indonesia	Madagascar
Croatia	Ecuador	Kenya	Malawi
Curaçao	Equatorial Guinea	Kiribati	Mali
Cyprus	Fiji	Kosovo	Mozambique
Czech Republic	Gabon	Kyrgyz Republic	Nepal
Denmark	Grenada	Lao PDR	Niger
Estonia	Guatemala	Lesotho	Rwanda
Faroe Islands	Guyana	Mauritania	Senegal
Finland	Iran, Islamic Rep.	Micronesia, Fed. Sts.	Sierra Leone
France	Iraq	Moldova	Somalia
French Polynesia	Jamaica	Mongolia	South Sudan
Germany	Jordan	Morocco	Syrian Arab Republic
Gibraltar	Kazakhstan	Myanmar	Tajikistan
Greece	Lebanon	Nicaragua	Tanzania
Greenland	Libya	Nigeria	Togo
Guam	Macedonia, FYR	Pakistan	Uganda
Hong Kong SAR, China	Malaysia	Papua New Guinea	Yemen, Rep.
Hungary	Maldives	Philippines	Zimbabwe
Iceland	Marshall Islands	São Tomé and Principe	
Ireland	Mauritius	Solomon Islands	
Isle of Man	Mexico	Sri Lanka	
Israel	Montenegro	Sudan	
Italy	Namibia	Swaziland	
Japan	Nauru	Timor-Leste	
Korea, Rep.	Paraguay	Tunisia	
Kuwait	Peru	Ukraine	
Latvia	Romania	Uzbekistan	
Liechtenstein	Russian Federation	Vanuatu	

Lithuania	Samoa	Vietnam	
Luxembourg	Serbia	West Bank and Gaza	
Macao SAR, China	South Africa	Zambia	
Malta	St. Lucia		
Monaco	St. Vincent and the Grenadines		
Netherlands	Suriname		
New Caledonia	Thailand		
New Zealand	Tonga		
Northern Mariana Islands	Turkey		
Norway	Turkmenistan		
Oman	Tuvalu		
Palau	Venezuela, RB		
Panama			
Poland			
Portugal			
Puerto Rico			
Qatar			
San Marino			
Saudi Arabia			
Seychelles			
Singapore			
Sint Maarten (Dutch part)			
Slovak Republic			
Slovenia			
Spain			
St. Kitts and Nevis			
St. Martin (French part)			
Sweden			
Switzerland			
Taiwan, China			

Trinidad and Tobago			
Turks and Caicos Islands			
United Arab Emirates			
United Kingdom			
United States			
Uruguay			
Virgin Islands (U.S.)			

Table 3.3: Classification of Countries by Global Burden of Disease Super-Regions

Central Europe, Eastern Europe, and Central Asia	High-income	Latin America and Caribbean	North Africa and Middle East	South Asia	Southeast Asia, East Asia, and Oceania	Sub-Saharan Africa
Albania	Andorra	Antigua	Afghanistan	Bangladesh	Am Samoa	Angola
Armenia	Argentina	Barbados	Algeria	Bhutan	Cambodia	Benin
Azerbaijan	Australia	Belize	Bahrain	India	China	Botswana
Belarus	Austria	Bermuda	Egypt	Nepal	Micronesia	Burkina Faso
Bosnia	Belgium	Bolivia	Iran	Pakistan	Fiji	Burundi
Bulgaria	Brunei	Brazil	Iraq		Guam	Cameroon
Croatia	Canada	Colombia	Jordan		Indonesia	Cape Verde
Czech	Chile	Costa Rica	Kuwait		Kiribati	C African Rep
Estonia	Cyprus	Cuba	Lebanon		Laos	Chad
Georgia	Denmark	Dominica	Libya		Malaysia	Comoros
Hungary	Finland	Dominican Rep	Morocco		Maldives	Congo
Kazakhstan	France	Ecuador	Oman		Marshall	Cote d'Ivoire
Kyrgyzstan	Germany	El Salvador	Palestine		Mauritius	Congo DR
Latvia	Greece	Grenada	Qatar		Myanmar	Djibouti
Lithuania	Greenland	Guatemala	Saudi Arabia		N Korea	Eq Guinea
Macedonia	Iceland	Guyana	Sudan		N Mariana	Eritrea
Moldova	Ireland	Haiti	Syria		PNG	Ethiopia
Mongolia	Israel	Honduras	Tunisia		Philippines	Gabon
Montenegro	Italy	Jamaica	Turkey		Samoa	Ghana
Poland	Japan	Mexico	UAE		Seychelles	Guinea
Romania	Luxembourg	Nicaragua	Yemen		Solomon	Guinea-Bissau
Russia	Malta	Panama			Sri Lanka	Kenya
Serbia	Netherlands	Paraguay			Taiwan	Lesotho
Slovakia	New Zealand	Peru			Thailand	Liberia
Slovenia	Norway	Puerto Rico			Timor-Leste	Madagascar
Tajikistan	Portugal	St Lucia			Tonga	Malawi
Turkmenistan	Singapore	St Vincent			Vanuatu	Mali
Ukraine	S Korea	Suriname			Vietnam	Mauritania
Uzbekistan	Spain	Bahamas				Mozambique

	Sweden	Trinidad Tobago				Namibia
	Switzerland	Venezuela				Niger
	UK	Virgin Isl US				Nigeria
	US					Rwanda
	Uruguay					Sao Tome Principe
						Senegal
						Sierra Leone
						Somalia
						S Africa
						S Sudan
						Swaziland
						Tanzania
						Gambia
						Togo
						Uganda
						Zambia
						Zimbabwe

Table 3.5. Global Burden of Disease Cause List

1. **Communicable Diseases**

**HIV/AIDS and tuberculosis**		
	Tuberculosis	
		Drug-susceptible tuberculosis
		Multidrug-resistant tuberculosis without extensive drug resistance
		Extensively drug resistant tuberculosis
	HIV/AIDS	
		Drug-susceptible HIV/AIDS - Tuberculosis
		Multidrug-resistant HIV/AIDS - Tuberculosis without extensive drug resistance
		Extensively drug resistant HIV/AIDS - Tuberculosis
		HIV/AIDS resulting in other diseases
**Diarrhea, lower respiratory, and other common infectious diseases**		
	Diarrheal diseases	
		Cholera
		Other salmonella infections
		Shigellosis
		Enteropathogenic E coli infection
		Enterotoxigenic E coli infection
		Campylobacter enteritis
		Amoebiasis
		Cryptosporidiosis
		Rotaviral enteritis

		Aeromonas
		Clostridium difficile
		Norovirus
		Adenovirus
		Other bacterial foodborne diarrhea
		Other diarrheal diseases
	Intestinal infectious diseases	
		Typhoid fever
		Paratyphoid fever
		Other intestinal infectious diseases
	Lower respiratory infections	
		Influenza
		Pneumococcal pneumonia
		H influenzae type B pneumonia
		Respiratory syncytial virus pneumonia
		Other lower respiratory infections
	Upper respiratory infections	
	Otitis media	
	Meningitis	
		Pneumococcal meningitis
		H influenzae type B meningitis
		Meningococcal meningitis
		Other meningitis
	Encephalitis	
	Diptheria	
	Whooping cough	
	Tetanus	
	Measles	
	Varicella and herpes zoster	
**Neglected tropical diseases and malaria**		
	Malaria	
	Chagas disease	
	Leishmaniasis	
		Visceral leishmaniasis
	African trypanosomiasis	
	Schistosomiasis	
	Cysticercosis	
	Cystic echinococcosis	
	Dengue	
	Yellow fever	
	Rabies	
	Intestinal nematode infections	
		Ascariasis

	Ebola	
	Zika virus	
	Other neglected tropical diseases	
**Maternal disorders**		
	Maternal hemorrhage	
	Maternal sepsis and other maternal infections	
	Maternal hypertensive disorders	
	Maternal obstructed labor and uterine rupture	
	Maternal abortion, miscarriage, and ectopic pregnancy	
	Indirect maternal deaths	
	Late maternal deaths	
	Maternal deaths aggravated by HIV/AIDS	
	Other maternal disorders	
**Neonatal disorders**		
	Neonatal preterm birth complications	
	Neonatal encephalopathy due to birth asphyxia and trauma	
	Neonatal sepsis and other neonatal infections	
	Hemolytic disease and other neonatal jaundice	
	Other neonatal disorders	
**Nutritional deficiencies**		
	Protein-energy malnutrition	
	Iodine deficiency	
	Iron-deficiency anemia	
	Other nutritional deficiencies	
**Other communicable, maternal, neonatal, and nutritional diseases**		
	Sexually transmitted diseases excluding HIV	
		Syphilis
		Chlamydial infection
		Other sexually transmitted diseases
	Hepatitis	
		Acute hepatitis A
		Hepatitis B
		Hepatitis C
		Acute hepatitis E
	Other infectious diseases	

2. Non-communicable Diseases

**Neoplasms**	
	Lip and oral cavity cancer
	Nasopharynx cancer
	Other pharynx cancer
	Esophageal cancer

	Stomach cancer	
	Colon and rectum cancer	
	Liver cancer	
		Liver cancer due to hepatitis B
		Liver cancer due to hepatitis C
		Liver cancer due to alcohol use
		Liver cancer due to other causes
	Gallbladder and biliary tract cancer	
	Pancreatic cancer	
	Larynx cancer	
	Tracheal, bronchus, and lung cancer	
	Malignant skin melanoma	
	Non-melanoma skin cancer	
		Non-melanoma skin cancer (squamous-cell carcinoma)
		Non-melanoma skin cancer (basal cell carcinoma)
	Breast cancer	
	Cervical cancer	
	Uterine cancer	
	Ovarian cancer	
	Prostate cancer	
	Testicular cancer	
	Kidney cancer	
	Bladder cancer	
	Brain and nervous system cancer	
	Thyroid cancer	
	Mesothelioma	
	Hodgkin lymphoma	
	Non-Hodgkin lymphoma	
	Multiple myeloma	
	Leukemia	
		Acute lymphoid leukemia
		Chronic lymphoid leukemia
		Acute myeloid leukemia
		Chronic myeloid leukemia
	Other neoplasms	
**Cardiovascular diseases**		
	Rheumatic heart disease	
	Ischemic heart disease	
	Cerebrovascular disease	

		Ischemic stroke
		Hemorrhagic stroke
	Hypertensive heart disease	
	Cardiomyopathy and myocarditis	
	Atrial fibrillation and flutter	
	Aortic aneurysm	
	Peripheral vascular disease	
	Endocarditis	
	Other cardiovascular and circulatory diseases	
**Chronic respiratory diseases**		
	Chronic obstructive pulmonary disease	
	Pneumoconiosis	
		Silicosis
		Asbestosis
		Coal workers pneumoconiosis
		Other pneumoconiosis
	Asthma	
	Interstitial lung disease and pulmonary sarcoidosis	
	Other chronic respiratory diseases	
**Cirrhosis and other chronic liver diseases**		
	Cirrhosis and other chronic liver diseases due to hepatitis B	
	Cirrhosis and other chronic liver diseases due to hepatitis C	
	Cirrhosis and other chronic liver diseases due to alcohol use	
	Cirrhosis and other chronic liver diseases due to other causes	
**Digestive diseases**		
	Peptic ulcer disease	
	Gastritis and duodenitis	
	Appendicitis	
	Paralytic ileus and intestinal obstruction	
	Inguinal, femoral, and abdominal hernia	
	Inflammatory bowel disease	
	Vascular intestinal disorders	
	Gallbladder and biliary diseases	
	Pancreatitis	
	Other digestive diseases	
**Neurological disorders**		
	Alzheimer disease and other dementias	
	Parkinson disease	
	Epilepsy	
	Multiple sclerosis	
	Motor neuron disease	

	Tension-type headache	
	Medication overuse headache	
	Other neurological disorders	
**Mental and substance use disorders**		
	Schizophrenia	
	Alcohol use disorders	
	Drug use disorders	
		Opioid use disorders
		Cocaine use disorders
		Amphetamine use disorders
		Cannabis use disorders
		Other drug use disorders
	Depressive disorders	
		Major depressive disorder
		Dysthymia
	Bipolar disorder	
	Anxiety disorders	
	Eating disorders	
		Anorexia nervosa
		Bulimia nervosa
	Autistic spectrum disorders	
		Autism
		Asperger syndrome
	Attention-deficit/hyperactivity disorder	
	Conduct disorder	
	Idiopathic intellectual disability	
	Other mental and substance use disorders	
**Diabetes, urogenital, blood, and endocrine diseases**		
	Diabetes mellitus	
	Acute glomerulonephritis	
	Chronic kidney disease	
		Chronic kidney disease due to diabetes mellitus
		Chronic kidney disease due to hypertension
		Chronic kidney disease due to glomerulonephritis
		Chronic kidney disease due to other causes
	Urinary diseases and male infertility	
		Interstitial nephritis and urinary tract infections
		Urolithiasis
		Benign prostatic hyperplasia
		Male infertility due to other causes
		Other urinary diseases

	Gynecological diseases	
		Uterine fibroids
		Polycystic ovarian syndrome
		Female infertility due to other causes
		Endometriosis
		Genital prolapse
		Premenstrual syndrome
		Other gynecological diseases
	Hemoglobinopathies and hemolytic anemias	
		Thalassemias
		Thalassemia trait
		Sickle cell disorders
		Sickle cell trait
		G6PD deficiency
		G6PD trait
		Other hemoglobinopathies and hemolytic anemias
	Endocrine, metabolic, blood, and immune disorders	
**Musculoskeletal disorders**		
	Rheumatoid arthritis	
	Osteoarthritis	
	Low back and neck pain	
		Low back pain
		Neck pain
	Gout	
	Other musculoskeletal disorders	
**Other non-communicable diseases**		
	Congenital anomalies	
		Neural tube defects
		Congenital heart anomalies
		Orofacial clefts
		Down syndrome
		Turner syndrome
		Klinefelter syndrome
		Chromosomal unbalanced rearrangements
		Other chromosomal abnormalities
	Skin and subcutaneous diseases	
		Dermatitis
		Psoriasis
		Cellulitis

		Pyoderma
		Scabies
		Fungal skin diseases
		Viral skin diseases
		Acne vulgaris
		Alopecia areata
		Pruritus
		Urticaria
		Decubitus ulcer
		Other skin and subcutaneous diseases
	Sense organ diseases	
		Glaucoma
		Cataract
		Macular degeneration
		Uncorrected refractive error
		Other hearing loss
		Other vision loss
		Other sense organ diseases
	Oral disorders	
		Deciduous caries
		Permanent caries
		Periodontal diseases
		Edentulism and severe tooth loss
		Other oral disorders
	Sudden infant death syndrome	

3. Injuries

**Transport injuries**		
	Road injuries	
		Pedestrian road injuries
		Cyclist road injuries
		Motorcyclist road injuries
		Motor vehicle road injuries
		Other road injuries
	Other transport injuries	
**Unintentional Injuries**		
	Falls	
	Drowning	
	Fire, heat and hot substances	
	Poisonings	
	Exposure to mechanical forces	

		Unintentional firearm injuries
		Unintentional suffocation
		Other exposure to mechanical forces
	Adverse effects of medical treatment	
	Animal contact	
		Venomous animal contact
		Non-venomous animal contact
	Foreign body	
		Pulmonary aspiration and foreign body in airway
		Foreign body in other body part
	Environmental heat and cold exposure	
	Other unintentional injuries	
**Self-harm and interpersonal violence**		
	Self-harm	
		Self-harm by firearm
		Self-harm by other specified means
	Interpersonal violence	
		Physical violence by firearm
		Physical violence by sharp object
		Physical violence by other means
**Forces of nature, conflict and terrorism, executions and police conflict**		
	Exposure to forces of nature	
	Conflict and terrorism	
	Executions and police conflict	

Supplementary Methods Annex Works Cited

1. Global Burden of Disease Collaborative Network, Institute for Health Metics and Evaluation. Global Burden of Disease Study 2016 (GBD 2016) disability-adjusted life years and healthy life expectancy 1990-2016. 2017. http://ghdx.healthdata.org/record/global-burden-disease-study-2016-gbd-2016-disability-adjusted-life-years-and-healthy-life (accessed July 10, 2018).
2. Global Burden of Disease Collaborative Network, Institute for Health Metrics and Evaluation. Global Burden of Disease Study 2016 (GBD 2016) all-cause under-5 mortality, adult mortality, and life expectancy 1970-2016. 2017. http://ghdx.healthdata.org/record/global-burden-disease-study-2016-gbd-2016-all-cause-under-5-mortality-adult-mortality-and (accessed July 10, 2018).
3. Global Burden of Disease Collaborative Network, Institute for Health Metrics and Evaluation. Global Burden of Disease Study 2016 (GBD 2016) covariates 1980-2016. 2017. http://ghdx.healthdata.org/record/global-burden-disease-study-2016-gbd-2016-covariates-1980-2016 (accessed July 10, 2018).
4. GBD 2015 Risk Factors Collaborators. Global, regional, and national comparative risk assessment of 79 behavioural, environmental and occupational, and metabolic risks or clusters of risks, 1990–2015: a systematic analysis for the Global Burden of Disease Study 2015. *Lancet* 2016; **388:** 1659–1724.
5. Institute for Health Metrics and Evaluation (IHME). Development assistance for health database 1990-2017. Seattle: Institute for Health Metrics and Evaluation, 2017. http://www.healthdata.org/policy-report/financing-global-health-2017 (accessed July 10, 2018).
6. Dieleman JL, Haakenstad A, Micah A et al. Spending on health and HIV/AIDS: domestic health spending and development assistance in 188 countries, 1995–2015. *Lancet* 2018; **391:** 1799–829.
7. Global Burden of Disease Health Financing Collaborator Network. Global health spending 1995-2015. Seattle: Institute for Health Metrics and Evaluation, 2017.http://ghdx.healthdata.org/record/global-health-spending-1995-2015 (accessed July 10, 2018).
8. Global Burden of Disease Collaborative Network. Global Burden of Disease Study 2016 (GBD 2016) population estimates 1950-2016. Seattle: Institute for Health Metrics and Evaluation, 2017. http://ghdx.healthdata.org/record/global-burden-disease-study-2016-gbd-2016-population-estimates-1950-2016 (last accessed July 10, 2018).
9. Global Burden of Disease Collaborative Network. Global Burden of Disease Study 2016 (GBD 2016) socio-demographic index (SDI) 1970–2016. Seattle: Institute for Health Metrics and Evaluation, 2017. http://ghdx.healthdata.org/record/global-burden-disease-study-2016-gbd-2016-socio-demographic-index-sdi-1970%E2%80%932016 (accessed July 10, 2018).
10. Institute for Health Metrics and Evaluation (IHME). Global educational attainment 1970-2015. Seattle: Institute for Health Metrics and Evaluation, 2015. http://ghdx.healthdata.org/record/global-educational-attainment-1970-2015 (accessed July 10, 2018).
11. Institute for Health Metrics and Evaluation (IHME). Infant and child mortality estimates by country 1970-2010. Seattle: Institute for Health Metrics and Evaluation, 2010. http://ghdx.healthdata.org/record/infant-and-child-mortality-estimates-country-1970-2010 (accessed July 10, 2018).
12. Coppedge M, Gerring J, Knutsen CH et al. V-Dem [country-year/country-date] D=dataset v8. Var. Democr. V-Dem Proj. 2018. https://www.v-dem.net/en/data/data-version-8/ (accessed July 10, 2018).
13. Pemstein D, Marquardt KL, Tzelgov E, Wang Y, Krusell J, Miri F. The V-Dem measurement model: latent variable analysis for cross-national and cross-temporal expert-coded data. University of Gothenburg: Varieties of Democracy Institute, 2018. http://dx.doi.org/10.2139/ssrn.3167764 (accessed July 10, 2018).
14. Munck GL, Verkuilen J. Conceptualizing and measuring democracy: evaluating alternative indices. *Comp Polit Stud* 2002; **35**: 5–34.
15. Coppedge M, Gerring J, Altman D et al. Conceptualizing and measuring democracy: a new approach. *Perspect Polit* 2011; **9**: 247–67.
16. Dahl RA. Polyarchy: participation and opposition. Yale University Press, 1971.
17. Teorell J, Coppedge M, Skaaning SE, Lindberg SII. Measuring electoral democracy with V-Dem data: introducing a new polyarchy index. University of Gothenburg: Varieties of Democracy Institute, 2016.

18. Gerring J, Thacker SC, Alfaro R. Democracy and human development. *J Polit* 2012; **74**: 1–17.
19. United Nations Population Division. Urban population (% of total). The World Bank. 2018. https://data.worldbank.org/indicator/SP.URB.TOTL.IN.ZS (accessed July 10, 2018).
20. World Bank. World development indicators 2017. World Bank. 2017. https://openknowledge.worldbank.org/handle/10986/26447?locale-attribute=en (accessed July 10, 2018).
21. Nunn N, Puga D. Ruggedness: the blessing of bad geography in Africa. *Rev Econ Stat* 2012; **94**: 20–36.
22. Abadie A, Gardeazabal J. The economic costs of conflict: a case study of the Basque country. *Am Econ Rev* 2003; **93**: 113–32.
23. Abadie A, Diamond A, Hainmueller J. Synthetic control methods for comparative case studies: estimating the effect of California's tobacco control program. *J Am Stat Assoc* 2010; **105**: 493–505.
24. Cheibub JA, Gandhi J, Vreeland JR. Democracy and dictatorship revisited. *Public Choice* 2009; **143**: 67–101.
25. Blaydes L, Kayser MA. Counting calories: democracy and distribution in the developing world. *Int Stud Q* 2011; **55:** 887–908.
26. Abadie A, Diamond A, Hainmueller J. Comparative politics and the synthetic control method. *American Journal of Political Science*. 2015; **59:** 495-510.
27. Galiani S, Quistorff B. The synth_runner package: utilities to automate synthetic control estimation using synth. *Stata J* 2017; **17**: 834–49.
28. Cavallo E, Galiani S, Noy I, Pantano J. Catastrophic natural disasters and economic growth. *Rev Econ Stat* 2013; **95**: 1549–61.
29. Groemping U, Matthias L. Package 'relaimpo': relative importance of regressors in linear models 2010, version 2.2-3. 2018. https://cran.r-project.org/web/packages/relaimpo/relaimpo.pdf (accessed July 9, 2018).
30. Greene WH. Econometric analysis. Upper Saddle River: Prentice Hall, 2012.
31. Lindeman RH, Merenda PF, Gold RZ. Introduction to bivariate and multivariate analysis. Glenview, IL: Scott, Foresman and Company, 1980.
32. Dieleman JL, Templin T, Sadat N, et al. National spending on health by source for 184 countries between 2013 and 2040. *Lancet* 2016; **387:** 2521–35.
